# The intestinal microbial community and function of *Riptortus pedestris* at different developmental stages and its effects on development

**DOI:** 10.3389/fmicb.2025.1517280

**Published:** 2025-01-22

**Authors:** Yanbin Wang, Rong Li, Chunjing Wang, Ting Sun, Hongjuan Zhang, Fang Zhao, Jiehui Liu, Yuqiong Hao, Xiansheng Xie

**Affiliations:** ^1^Institute of Wheat Research, Shanxi Agricultural University, Linfen, Shanxi, China; ^2^College of Plant Protection, Shanxi Agricultural University, Taigu, Shanxi, China; ^3^Key Laboratory of Sustainable Dryland Agriculture (Coconstruction by Ministry and Province) Ministry of Agriculture and Rural Affairs, Institute of Wheat Research, Shanxi Agricultural University, Linfen, Shanxi, China

**Keywords:** *Riptortus pedestris*, gut microbiota, microbial function, antibiotic resistance genes, development

## Abstract

**Introduction:**

*Riptortus pedestris* is a destructive pest that threatens multiple leguminous crops in China. The intestinal microbiota plays a crucial role in the growth and reproduction of host insects. However, the composition and function of the gut microbiota at different developmental stages remain unclear.

**Methods:**

Here, metagenomic sequencing was performed to clarify the gut microbial diversity and function in 2nd-, 3rd-, 4th-, and 5th- instar nymphs (2 N–5 N) and female adults (FAs) of *R. pedestris* and the effects of vital gut bacteria on development was detected. The gut bacteria have the stage specificity, indicating their function in the development of *R. pedestris*.

**Results:**

*Enterococcus* and *Caballerronia* were the predominant bacteria present during the development of the 2 N–FAs. In addition, the microbial abundances in the 3 N and 4 N guts were significantly greater than those in the others guts. Furthermore, 5 N harbored the abundant microbiota *Burkholderia*-*Paraburkholderia*-*Caballeronia*. The metabolic pathways were significantly enriched from 2 N to FAs. Carbohydrate metabolism, including glycoside hydrolases (GHs) and glycosyl transferases (GTs), occurs throughout the entire developmental stage. Many antibiotic resistance genes (ARGs) were detected from 2 N to FAs. The bacteria from Pseudomonadota and Bacillota presented a broad spectrum of antibiotic resistance. Excitingly, *Burkholderia* bacteria eliminated by antibiotic treatment were unable to molt normally, and their lifespan was shortened in nymphs, indicating that the gut microbiota had a significant effect on nymph development.

**Conclusion:**

In summary, our results, for the first time, systematically illustrate the abundance and function across the gut microbiota from the different developmental stages of *R. pedestris* and demonstrate that the genera *Burkholderia* are crucial during the development of *R. pedestris*. This study provides the basis for stinkbug management strategies that focus on the pivotal gut microbiota.

## 1 Introduction

The gut microbiota plays an important role in the evolution and reproduction of insects, as well as in maintaining ecosystem balance (Schoeler and Caesar, 2019; Shamjana et al., 2024). These microorganisms are usually able to provide nutrients and new energy pathways that are difficult for the host to obtain, allowing them to reproduce in habitats that were originally unfavorable for their survival (Shamjana et al., 2024). At present, the intestine is the organ with the most microbial colonization in insects, as the intestinal mucosa has a large surface area that can accommodate many microbial communities to colonize its surface (Sekirov et al., 2010). The gut microbiota typically participates in regulating various physiological functions of host insects, such as promoting insect fitness by synthesizing essential nutrients, increasing host insecticide resistance, and even protecting insects from natural enemies or pathogens (Engel and Moran, 2013; Jang and Kikuchi, 2020). For example, the gut microbiota of honeybees not only affects their reproductive ability but also influences their learning and memory behavior by regulating tryptophan metabolism (Zhang Z. et al., 2022).

The diversity of the gut microbiota in insects varies greatly at different stages due to various factors, such as the habitat environment, food source, and host type, as well as coevolution with the host (Engel and Moran, 2013; Yun et al., 2014; Ng et al., 2018). In addition, in completely metamorphosed insects, the intestinal tissue undergoes complete changes and even complete elimination. However, many insects have specialized crypt structures in their intestines that can promote the retention of microorganisms. For example, bacteria belonging to the *Burkholderia* genus settle in the M4 region of the gut of the stinkbug, which is composed of a double row of crypt structures (Kikuchi et al., 2005). *Enterobacter* and *Enterococcus* are the most dominant genera in 4th- and 6th-instar larvae, female pupae, male pupae, female adults and male adults in *Spodoptera frugiperda*, whereas *Ralstonia* and *Sediminibacterium* are the most abundant in eggs (Fu et al., 2023). By controlling the gut microbiota of insects, the adaptability of host insects to plants can be reduced, which provides the possibility for developing new and efficient insecticidal strategies. For example, in the *Adelphocoris suturalissome*, the intestinal bacteria *Serratia marcescens*, which has direct insecticidal effects, can be used as a biological control agent (Preuß et al., 2019; Luo et al., 2021; Zhang et al., 2023). In addition, symbiotic bacteria-mediated RNAi-targeted insecticidal technology can be developed to use highly efficient and specific volatile substances produced by intestinal bacteria to attract or expel pests (Elston et al., 2023). In addition, the study of the insect gut microbiota involves the exploration of microbial resources with special functions. By isolating and purifying the gut microbiota, microbial strains with important application value, such as strains that produce active substances, antibacterial agents, and special enzymes, can be obtained (Shao et al., 2017; Gao et al., 2021). These studies not only contribute to the development of new bioactive substances but also provide new ideas and methods for the biological control of pests.

The soybean stinkbug *Riptortus pedestris* is the main pest affecting leguminous crops in the Huang-Huai-Hai region of China (Li et al., 2019; Zhang H. et al., 2022). They mainly gather and suck sap from stems and pods through 1st–5th instar nymphs and adults (Nakajima et al., 2010). Their invasion causes crops to be unable to shed leaves normally, and most pods shrink during maturity, which is known as “stay-green syndrome” (Wei et al., 2023). This phenomenon has caused a serious reduction in the yield of leguminous crops, even resulting in no harvest at all (Li et al., 2019). Therefore, the main problem faced in legume production is how to prevent and control the harm caused by soybean stinkbugs effectively. The structure and function of the gut microbiota are crucial for the growth and development of different stages of the stinkbugs.

Resistance genes are widely present in both the insect gut microbiota and other environmental microorganisms. The overuse of antibiotics leads to irreversible changes in microbial communities in the insect body and environment, enhancing the antibiotic resistome of insects. Therefore, research on resistance genes has received widespread attention from researchers (Martínez et al., 2015). Many studies have reported that the gut microbiota is resistant to antibiotics. For example, *Enterococcus* strains present different levels and types of resistance to vancomycin (Courvalin, 2006). The gut microbiome in animals and insects is frequently enriched in subtypes of tetracyclines, such as *tetQ*, *tetO*, and *tetM* (Yang et al., 2022). Therefore, the identification of antibiotic genotypes could help us assess antibiotic tolerance and identify effective antibiotics to control pests.

In recent years, many studies have investigated the gut symbiotic *Burkholderia* of *R. pedetris.* This symbiont enhances the host’s reproductive capacity, drug resistance, and immunity while reducing the host’s developmental duration (Kikuchi et al., 2011, 2012; Kim et al., 2015a). However, these studies focused mainly on the gut microbial function associated with a certain stage in *R. pedetris.* The lifecycle of the soybean stinkbug includes eggs, 1st–5th instar nymphs, female adults, and male adults. The composition and function of the gut microbiota at different developmental stages have yet to be reported. With the development of high-throughput sequencing technology and omics technology, the mining and application of insect gut metagenomic big data have become popular research topics, greatly promoting the ability of human microbial resource utilization. This study used metagenomic sequencing to analyse the composition and function of the gut microbiota in 2nd–5th instar nymphs and female adults of *R. pedestris* and explored the application prospects of major microorganisms in the prevention and control of *R. pedestris*. Furthermore, the effects of antibiotic treatment on the development of stinkbug were studied. Hence, the results of our study will not only contribute to the understanding of some key roles of the gut microflora in the different development stages of *R. pedestris* but also provide insights into the management of this pest.

## 2 Materials and methods

### 2.1 Insect rearing and plants

*Riptortus pedestris* colony (mtCOI GenBank accession no. OR654302) was collected at the Institute of Wheat Research, Shanxi Agricultural University, China, in 2022 and 2023. The colony was maintained on soybean plants (*Glycine max* L. cv. Jindou 36). The *R. pedestris* colony harbors a few gut symbiotic bacteria, such as *Burkholderia* sp. (Takeshita et al., 2014), *Caballeronia insecticola* (Jouan et al., 2024), and *Serratia marcescens* (Lee and Lee, 2022).

The soybean plants were grown in a potting mix. Two or three soybean plants were grown in 1.5 L pots to the six-to-seven true-leaf stage to raise *R. pedestris*. All *R. pedestris* colonies and soybean plants were maintained in climate-controlled chambers at 25 ± 2°C with a 16 h light: 8 h dark regime and 50–60% relative humidity (RH). The LED fluorescent lights were used, and the light intensity in the walk-in chamber was approximately 400 μmol/m^2^ s.

### 2.2 Sample collection

**Figure 1:**
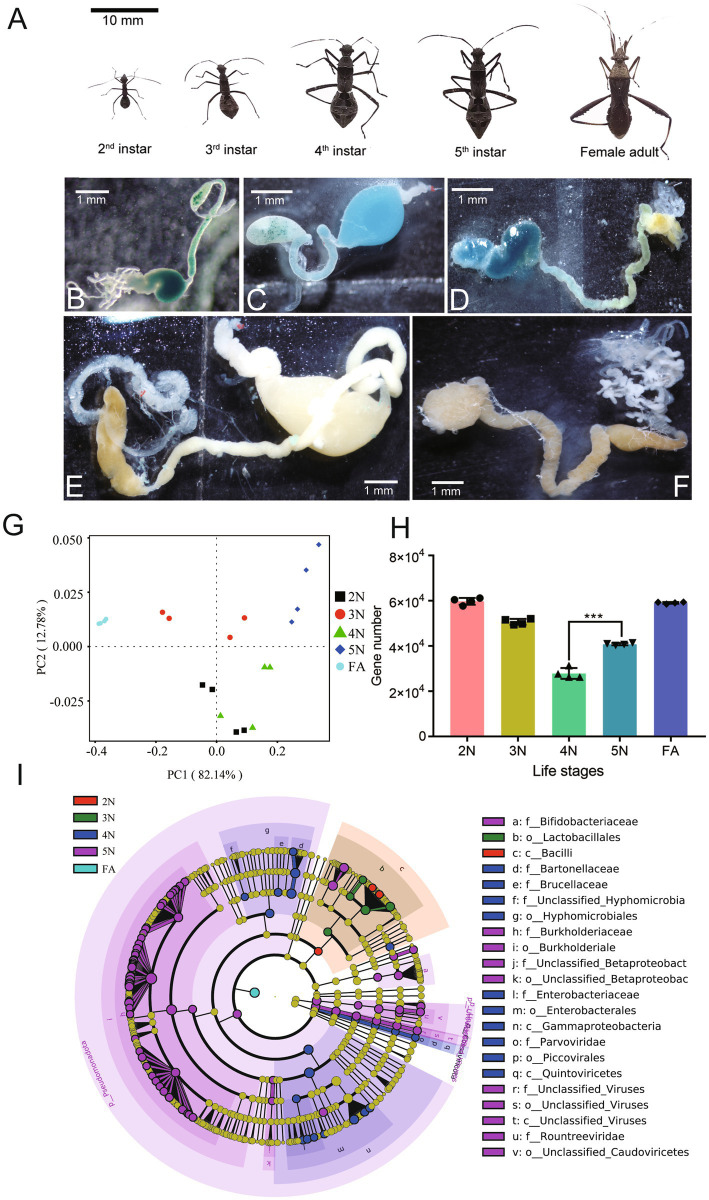
Differential intestinal species of *Riptortus pedestris* at different developmental stages. **(A–F)** Stinkbugs **(A)** and corresponding intestinal samples **(B–F)** from different developmental stages were collected. Note the midgut 1st section (M1) is filled with a large amount of egg yolk whose color changes from green to dark blue. **(A)** Scale bar: 10 mm; **(B–F)** Scale bar: 1 mm. **(G)** Community structure similarity of the gut microbiota among different developmental stages in *R. pedestris* according to PCoA based on the Bray–Curtis distance. **(H)** Analysis of differences in the number of species-related genes between groups. **(I)** Evolutionary branching diagram of different species. The circle radiating from the inside out represents the classification level from phylum to genus (or species). Each small circle at a different classification level represents a classification at that level, and the diameter of the small circle is proportional to the relative abundance. Species with no significant differences are uniformly colored yellow, whereas biomarkers of different species follow the group for coloring. Red nodes represent microbial communities that play important roles in the red group, whereas green nodes represent microbial communities that play important roles in the green group. The species names represented by English letters in the figure are shown in the legend on the right. 2 N, 2nd instar nymph; 3 N, 3rd instar nymph; 4 N, 4th instar nymph; 5 N, 5th instar nymph; FA, female adult.

Newly hatched 2nd, 3rd, 4th, and 5th nymphs and female adults were collected and divided into G1, G2, G3, G4, and G5 (Figure 1A; Supplementary Table S1). These samples’ surface were sterilized in 70% ethanol, and then the midguts of each sample were dissected in phosphate-buffered saline (1 × PBS) (Figures 1B–F), immediately stored in liquid nitrogen for ten minutes and then stored at −80°C for further analysis and metagenomic sequencing. Three biological replicates were performed for each group.

### 2.3 Genomic DNA extraction and metagenomic sequencing analysis

Five groups of samples were sent to Novogene Bioinformatics Technology Co. Ltd. (Beijing, China) for shotgun metagenome sequencing. Every group of samples was homogenized, and DNA was extracted according to the manufacturer’s recommended protocol for the QIAamp^®^ DNA Microbiome Kit (Cat. No. 51704, QIAGEN, Germany). This kit removes the host DNA to avoid affecting the sequencing result. The extracted gut genomic DNA was randomly sheared into 350 bp fragments, and sequencing libraries were generated. The obtained fragments were end-repaired, A-tailed and further ligated with an Illumina adapter. The fragments with adapters were PCR amplified, size selected, and purified. The library quality was checked with a Qubit 3.0 fluorometer (Life Technologies; Grand Island, NY, United States), real-time PCR was used for quantification, and a bioanalyzer was used for size distribution detection. The quantified libraries were pooled and sequenced on the Illumina NovaSeq X Plus platform (Illumina; San Diego, CA, United States) and the Agilent 2,100 (Agilent, Santa Clara, CA) system, according to the effective library concentration and data amount needed.

Metagenomic sequencing analysis was performed as described by Zhang Z. et al. (2022). Briefly, 20 metagenomic DNA libraries were constructed via paired-end 150 bp (PE150) sequencing. Considering the possibility of host contamination in samples, clean data needs to be mapped to the host database to filter out reads that may come from host origin via Bowtie2 software (Karlsson et al., 2013).

### 2.4 Bioinformatics analysis

#### 2.4.1 Preprocessing of sequencing results

Readfq was used for preprocessing raw data from the Illumina sequencing platform to obtain clean data for subsequent analysis. Bowtie2 software is used with the following parameter settings: --end-to-end, −-sensitive, -I 200, and -X 400 (Karlsson et al., 2012, 2013; Scher et al., 2013).

#### 2.4.2 Assembly and prediction of the metagenome

Metagenome assembly analysis and prediction for the clean data were performed via MEGAHIT 11.2 (Li et al., 2015) and MetaGeneMark (v 3.38) (Yan et al., 2024) software, respectively. With the default parameters, MetaGeneMark was used to perform ORF prediction for the scaftigs (≥ 500 bp) of each sample, and the information with a length of less than 100 nt in the prediction results was filtered out (Sunagawa et al., 2015). CD-HIT was subsequently employed to obtain nonredundant ORFs with 95% identity and 90% coverage (Fu et al., 2012). Clean data from each sample were aligned to the initial gene catalogue by using Bowtie2 to calculate the number of reads of the genes in each sample alignment. Genes with <= 2 reads in each sample were filtered out to finally determine the gene catalogue (unigenes) for subsequent analysis (Zeller et al., 2014).

### 2.5 Analysis of the gut microbial composition and diversity

The richness and evenness of the gut bacteria were estimated via alpha diversity, including the Ace, Chao1, Shannon and Simpson indices, via QIIME 2 (Fung et al., 2021). Differences in the bacterial composition among the different developmental stages were calculated via principal coordinate analysis (PCoA) via nonparametric testing. Linear discriminant analysis effect size (LEfSe) (LDA score = 4, *p* < 0.05) was used to identify biomarkers with significantly different species between groups (Segata et al., 2011). Bar plots and heatmaps were generated for bacterial diversity and gene number differences in the different developmental stages via the R (version 4.0.3) package ggplot and pheatmap. To evaluate statistically significant differences between the groups, one-way ANOVA was performed with GraphPad Prism 7 at the 0.05 level.

### 2.6 Functional prediction of the gut microbiota

The potential metabolic functions of the gut bacterial communities were predicted via DIAMOND (Feng et al., 2015). The commonly used functional databases currently include the Kyoto Encyclopedia of Genes and Genomes (KEGG), evolutionary genealogy of genes: Nonsupervised Orthologous Groups (eggNOG), and Carbohydrate-Active enzymes Database (CAZy). To predict the functions of the gut microbiota communities in each developmental stage, the Kyoto Encyclopedia of Genes and Genomes (KEGG) and carbohydrate-active enzymes (CAZy) databases were used to predict microbial functions and functional pathways.

### 2.7 Antibiotic resistance gene abundance in the gut microbiota

Resistance genes are commonly present in the gut microbiota. Therefore, research on resistance genes has received widespread attention (Martínez et al., 2015). To further investigate the hazard mechanism of soybean stinkbugs, we conducted a statistical analysis of resistance genes at different developmental stages of *R. pedestris* via antibiotic resistance ontology (ARO) from the comprehensive antibiotic resistance database (CARD) (Jia et al., 2017).

### 2.8 Quantitative PCR and quantitative real-time PCR

**Figure 2:**
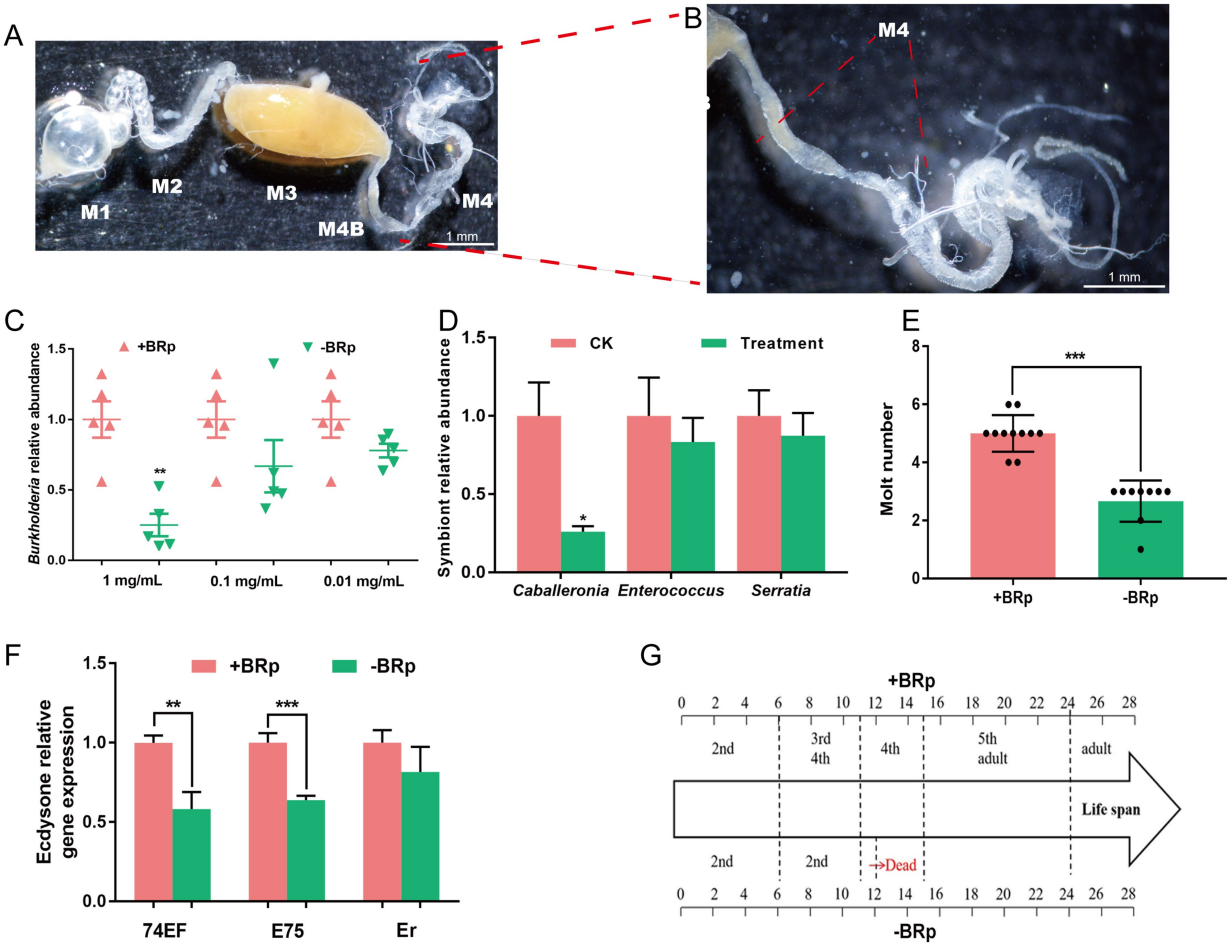
Effects of antibiotic treatment on the development of *R. pedestris*. **(A)** The gut of *R. pedestris* consists of five parts: M1, first midgut section; M2, second midgut section; M3, third midgut section; M4, fourth midgut section; and M4B, bulbous section before M4. **(B)** Enlarged images of M4 and M4B. M4 is a symbiotic organ composed of crypts. A and B, Scale bar: 1 mm. **(C)** Relative abundance of *Burkholderia* after treatment with different concentrations of tetracycline hydrochloride in +BRp and –BRp stinkbugs (*n* = 5). **(D)** Relative abundance of *Caballeronia*, *Enterococcus* and *Serratia* after the stinkbugs feeding on the distilled water containing 0.05% ascorbic acid supplemented with and without 1 mg/mL tetracycline hydrochloride for six days (*n* = 3). **(E)** Molt numbers of +BRp (*n* = 11) and –BRp (*n* = 9) stinkbugs. **(F)** Ecdysone relative gene expression of +BRp and −BRp (*n* = 3). **(G)** Life spans of +BRp (*n* = 11) and –BRp (*n* = 9). The error bars indicate the means ± SEs. Significant differences between +BRp and –BRp are indicated by asterisks: **, *p* < 0.01; ***, *p* < 0.001.

The 2nd-, 3rd-, 4th-, and 5th- instar nymphs and female adults were collected and then surface sterilized in 70% ethanol. Individual insects were subsequently dissected in 1× PBS, and a midgut region with crypts was carefully cut out (Figures 2A,B). The individuals or groups of insects were homogenized via a JXFSTPRP-CL-48 homogenizer (Shanghai Jingxin Industrial Development Co., Ltd.). DNA was extracted as described previously (Kikuchi et al., 2005) with some modifications. The DNA was then quantified via a Micro Drop spectrophotometer (Bio-DL, Shanghai, China). The abundance of the gut symbionts *Burkholderia*, *C. insecticola* (thereafter, *Caballeronia*), *Enterococcus* sp. (thereafter, *Enterococcus*) and *S. marcescens* (thereafter, *Serratia*) were quantified via qPCR using a LineGene 9,600 Fluorescent Quantitative PCR Detection System (Bioer, Hangzhou, China) with 2 × SYBR Green master mix (Selleck, United States) and their primers were shown in Supplementary Table S1. The qPCR primers were designed with Primer Premier 5.0 software (Premier Biosoft International, Palo Alto, CA, United States). The thermal cycling protocol included initial denaturation for 30 s at 95°C, followed by 40 cycles of 5 s at 95°C and 30 s at 60°C, with melting curve analysis confirming that only the specific products were amplified. Two technical replicates were performed for each of the 6 biological replicates for *R. pedestris.*

qRT–PCR was used to quantify the expression of 3 genes involved in the ecdysone-related pathway. Total RNA was extracted from the detected gut samples via the TRIzol reagent method (Mei5bio, Beijing). The RNA was then quantified via a Micro Drop spectrophotometer. The RNA was first-strand reverse transcribed via a Vazyme kit (HiScript III RT SuperMix for qPCR (+gDNA wiper), Nanjing, China). The qRT–PCR primers were designed with Primer Premier 5.0 software. The primers used for the ecdysone-related genes were shown in Supplementary Table S1. qRT–PCR was performed as follows: denaturation at 95°C for 5 min, followed by 40 cycles of 15 s at 95°C, 30 s at 60°C and 30 s at 72°C. Two technical replicates were performed for each of the three biological replicates.

Standard curves were constructed, and the amplification efficiency was estimated as described previously (Luan et al., 2015). The elongation factor 1 *α* (EF1α) gene of *R. pedestris* served as an endogenous control gene for normalization via qPCR and qRT–PCR (Kim et al., 2015b). The Primer was shown in Supplementary Table S1. The relative symbiont density and gene expression were calculated via the 2^−ΔCt^ method (Schmittgen and Livak, 2008).

### 2.9 Effects of the gut microbiota on development

To survey the influence of gut bacteria on host development, 2nd-instar nymphs were treated with tetracycline hydrochloride (Biotopped, Beijing, China) to eliminate the gut symbiont *Burkholderia*. The 2nd- instar nymphs were collected from the hatching cage, and released into each feeding chamber, and then fed distilled water containing 0.05% ascorbic acid solution (w/v) supplemented with tetracycline hydrochloride at 1, 0.1 or 0.01 mg/mL for 6 days (Treatment). The control insects were administered 0.05% ascorbic acid solution not supplemented with antibiotics (CK). The artificial diets were renewed every day. Following the diet regimens, these treated nymphs were transferred to soybean plants. The *R. pedestris* strains with reduced *Burkholderia* titres (-BRp) were obtained via antibiotic treatment, and the control *R. pedestris* strains (+BRp) were obtained via the addition of an ascorbic acid solution that was not supplemented with antibiotics. The molting and developmental periods were statistically analysed. To evaluate the significant differences between the different treatments, *t*-tests were performed with GraphPad Prism 7 at the 0.05 level.

## 3 Results

### 3.1 Analysis of the intestinal microbial metagenomic sequencing data and gene abundance in the different developmental stages of *Riptortus pedestris*

The guts from different developmental stages of *R. pedestris* (Figures 1A–F) were used for metagenomic sequencing. Twenty metagenomic DNA libraries constructed from the G1 to G5 groups were sequenced on the Illumina NovaSeq X Plus platform. As shown in Supplementary Table S1, there were 6,049.31–6,906.23 Mb of raw data from the G1 to G5 groups. After these data were filtered, 6,030.28–6,799.76 Mb of clean data were obtained from the G1 to G5 groups (Supplementary Table S1). The Q20s (%) of the G1 to G5 groups ranged from 98.10 to 99.03% (Supplementary Table S2). Furthermore, the GC contents (%) of the G1 to G5 groups ranged from 40.89 to 58.82% (Supplementary Table S2). From the G1 to G5 groups, the effective percentage (%) was greater than 98% in each group (Supplementary Table S2). A PCoA plot based on a phylum-level relative abundance profile revealed that axis 1 (PCoA1) explained 82.14% of the variability and that axis 2 (PCoA2) explained 12.78% of the variability. The PCoA plot revealed that the samples from different developmental stages were almost completely separated (Figure 1G). The above sufficient sequencing data indicated that these data were sufficient to estimate the gut bacterial diversity of *R. pedestris* fully.

We subsequently compared the number of genes associated with the gut microbiota at different developmental stages of *R. pedestris*. According to Spearman correlation coefficient analysis, gene abundance among samples was positively correlated, and there was a high correlation between different groups of samples (Supplementary Figure S1). In the five stages, the minimum number of genes was detected in the 4 N group compared with the other groups (Figure 1H, Wilcoxon rank-sum test, *p <* 0.001). However, there is no direct proportional relationship between the number of genes in a species and its richness. To screen for species with significant differences between groups, we conducted a phylogenetic analysis of the gut species between different groups via LEfSe at LDA > 4 and *p* < 0.05 (Figure 1I). In the 2 N and 3 N, the species richness was relatively limited. At the class level, Bacilli was more abundant in 2 N and at the order level, Lactobacillales was more ample in 3 N. In the 4 N and 5 N guts, there were individually 10 microbial communities at different taxonomic levels. At the order level, Hyphomicrobiales and Enterobacterales were more aboundant in G4 gut. At the family level, Burkholderiaceae was more plentiful in the 5 N midgut. Interestingly, the microbial communities in the FA intestine were concentrated at the phylum level (Figure 1I).

### 3.2 Diversity analysis of the gut microbiota of *Riptortus pedestris* at different stages

**Figure 3:**
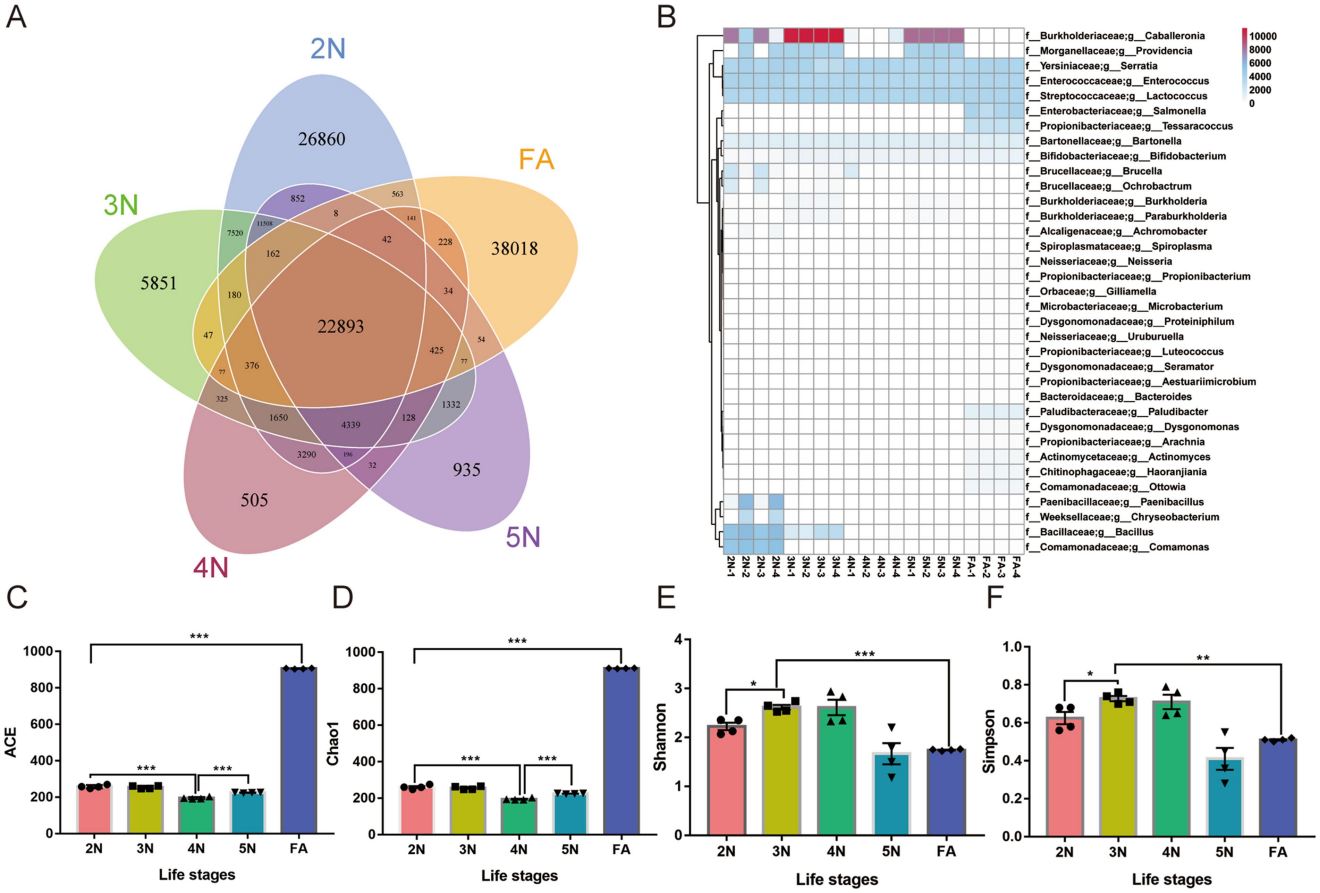
Analysis of the relative abundance and number of annotated genes and *α* diversity analysis of different groups of *R. pedestris.* **(A)** Unigene annotation count heatmap among different developmental stages in *R. pedestris.* **(B)** Venn diagrams depicting the number of genes common to and unique to different groups. **(C–F)** α diversity analysis of the gut microbiota at different life stages of *R. pedestris* by the ACE, Chao1, Shannon and Simpson indices. 2 N, 2nd instar nymph; 3 N, 3rd instar nymph; 4 N, 4th instar nymph; 5 N, 5th instar nymph; FA, female adult. Significant differences between groups are indicated by asterisks: *, *p* < 0.05; **, *p* < 0.01; ***, *p* < 0.001.

To further clarify the relative abundance of species in each sample, the abundances of gut microbial gene families and species diversity were quantified. The numbers of genes annotated from 2 N to FA were 26,860, 5,851, 505, 935, and 38,018, respectively, and 22,893 genes were collectively annotated (Figure 3A, Supplementary Table S4). Among all the annotated species, 3 genera had the highest abundance among the five groups, including *Serratia*, *Enterococcus and Lactococcus*. *Caballeronia and Providencia* presented high proportions of 2 N, 3 N and 5 N (Figure 3B).

The alpha diversity indices mainly include the Chao1 index, ACE index, Shannon index, and Simpson index. The ACE and Chao1 indices are commonly used to estimate unobserved species richness, whereas the Shannon and Simpson indices are used to measure species diversity. In the FAs, the number of unknown microorganisms was significantly greater than that in the G1 to G4 groups according to the ACE and Chao1 analyses (Figures 3C,D, *p* < 0.001; Supplementary Table S3), so the microbial community was relatively smaller than that in the other groups. According to Krona visualization analysis, up to 36% of the microorganisms in FA were unknown (Supplementary Figure S2E). However, according to the results of the Shannon and Simpson analyses, the microbial diversity of the 2 N–4 N groups were obviously greater than that of the other two groups (Figures 3E,F, *p* < 0.05; Supplementary Table S3). These results were consistent with the Krona visualization results (Supplementary Figures 2A–C).

**Figure 4:**
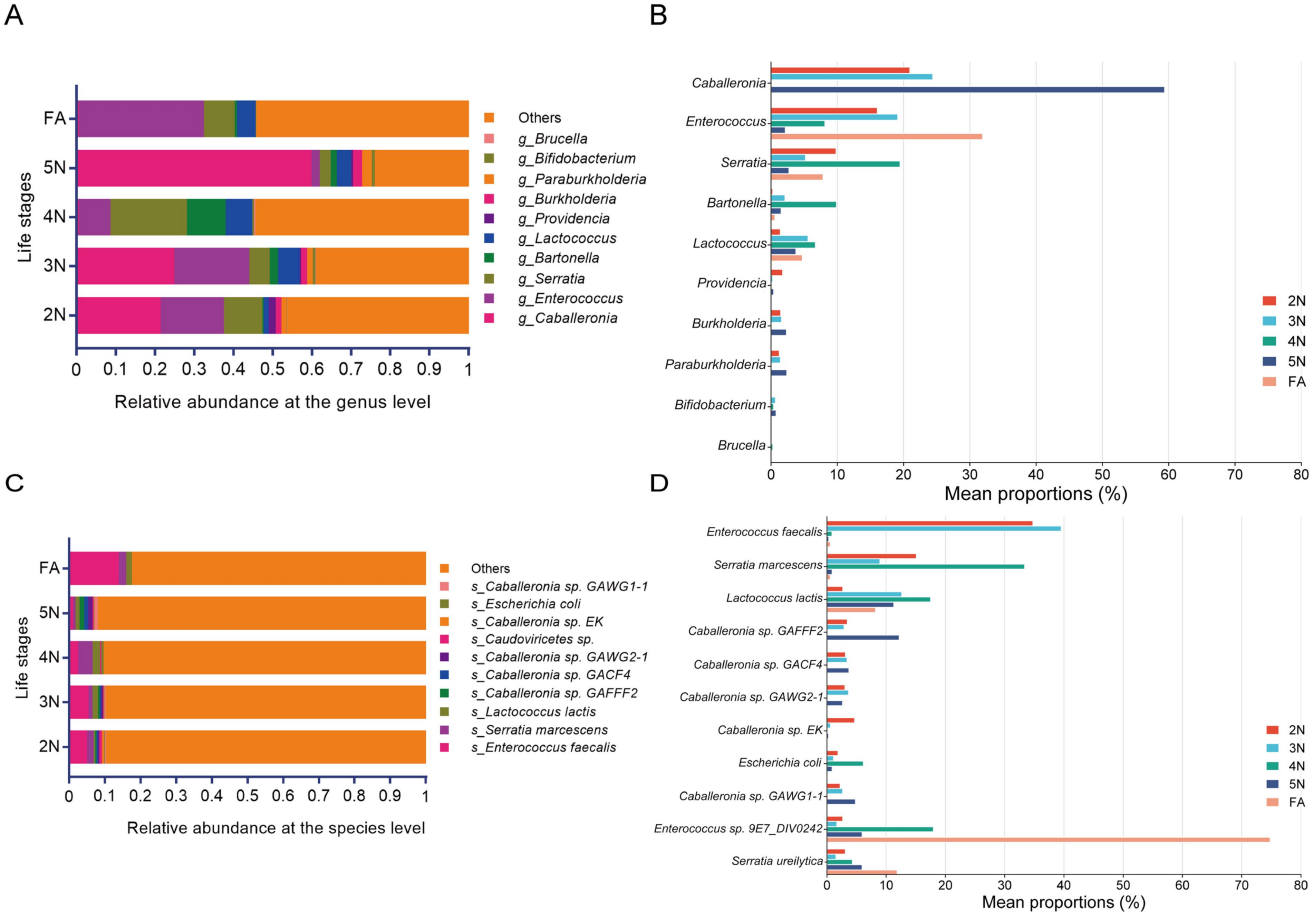
Compositional features of the entire gut microbiota of *R. pedestris* across different life stages. **(A,C)** Relative abundance of bacterial communities at the genus level **(A)** and species level **(C)**. **(B,D)** Relative abundances of the top 11 genera **(B)** and species **(D)** associated with significantly different developmental stages. Different colors represent different groups, and the color scale represents the percentage abundance of a certain genus **(B)** or species **(D)**. 2 N, 2nd instar nymph; 3 N, 3rd instar nymph; 4 N, 4th instar nymph; 5 N, 5th instar nymph; FA, female adult.

To investigate the composition of the *R. pedestris* bacterial community at different groups, the top 10 most abundant genera and species were analysed. At the genus level, the advantageous genera were *Caballeronia* and *Enterococcus*, followed by *Serratia* and *Lactococcus* at 2 N, 3 N, and 5 N. In contrast, *Caballeronia* and *Enterococcus* were significantly enriched in the 5 N and groups, respectively (Figure 4A; Supplementary Table S5). There were five genera whose relative abundances were dominant in different groups, namely, *Caballeronia, Enterococcus, Serratia, Bartonella and Lactococcus* (Figure 4B; Supplementary Table S6). At the species level, *Enterococcus faecalis* was significantly greater in the FA group than in the other groups (Figure 4C; Supplementary Tables S7, S8). The genus *Enterococcus* and the species *Enterococcus faecalis* were quite abundant in FA, and their proportions reached 34 and 14%, respectively (Supplementary Figure S2E). In addition, at both the genus and species levels, the number of bacteria in the gut microbiota was significantly lower in the FA stage than in the other developmental stages, but many unassigned bacteria were found (Figures 4A,C; Supplementary Figure S2E). In the other groups, *Enterococcus* sp., *Serratia* sp. and *Caballeronia* sp. were present in a significant proportion (Figure 4D), so these species could be biomarkers in the stinkbug gut. These results revealed that the microbial community members changed with the developmental stage of *R. pedestris*.

### 3.3 Key microbiotic species across different developmental stages

To clarify the predominant species at different developmental stages, linear discriminant analysis (LDA) effect size analysis (LEfSe) was performed to detect notable differences in the gut bacteria across the life stages of *R. pedetris*. The results of the rank sum test (LDA > 4, *p* < 0.05) revealed that the gut microbiota was the most abundant in the 5 N (Supplementary Figures 3A,B). On the basis of the heatmap analysis, *Burkholderia-Paraburkholderia-Caballeronia* was the predominant genus in the 5 N (Supplementary Figure 3B). Interestingly, compared with that in the other stages, *Burkholderia* density was greater in the 5 N stage (Supplementary Figure S4). Therefore, bacteria of the *Burkholderia* genus have great potential for application in the prevention and control of stinkbugs.

### 3.4 Functional prediction analysis of the gut microbiome between the different groups

**Figure 5:**
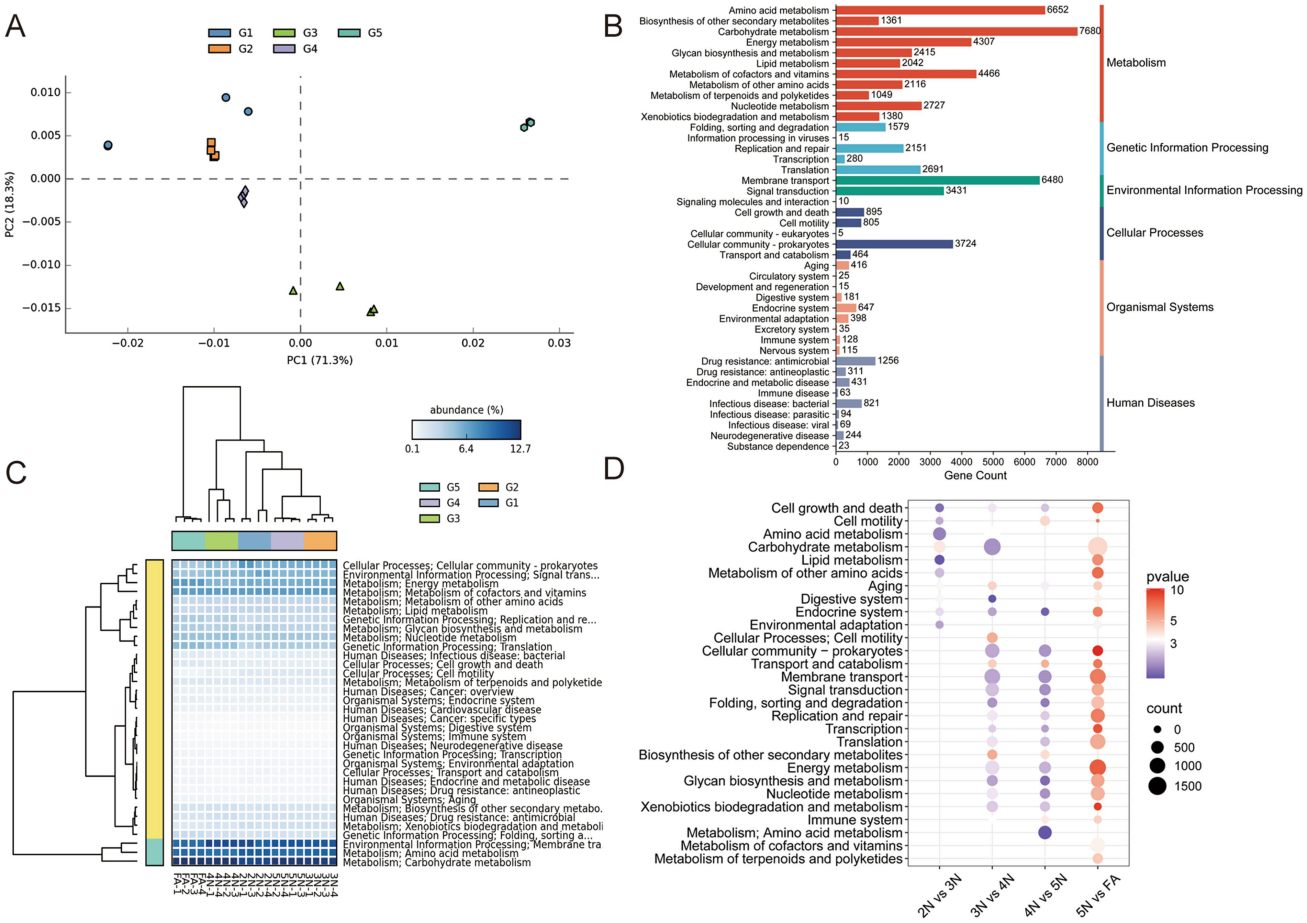
Functional enrichment and comparison of the gut microbiome associated with different developmental stages in *R. pedestris* at the KEGG pathway level 2. **(A)** PCA plot based on KEGG pathway level 2 of gut microbiome gene-family abundance. **(B)** Gene counts of KEGG pathway annotations at level 2. **(C)** Relative abundances of KEGG level 2 functions of the gut microbiome during the different developmental stages of *R. pedestris*. **(D)** KEGG analysis of metabolic pathways identified between every pair of stages. 2 N, 2nd instar nymph; 3 N, 3rd instar nymph; 4 N, 4th instar nymph; 5 N, 5th instar nymph; FA, female adult.

The abundances of microbial metabolic pathways were predicted via KEGG, and the functional differences in the gut microbiome were compared. A PCA plot based on gene metabolic function revealed that the samples enriched with 2 N to FA were almost completely spatially separated (Figure 5A). The results of the KEGG statistical analysis revealed that the most abundant genes in the gut microbiota of all the samples were associated with metabolism, particularly carbohydrate metabolism, amino acid metabolism, metabolism of cofactors and vitamins, energy metabolism and nucleotide metabolism (Figure 5B; Supplementary Table S9). These results revealed that the midgut-colonizing microbiota could provide essential amino acids and vitamins for the host, which are lacking in leguminous crops. In addition, genes involved in environmental information processing, including membrane transport and signal transduction, were largely enriched in the gut microbiome of all the samples (Figure 5B). This result further explained that the gut microbiota of the stinkbugs were obtained mainly from the surrounding environment. From 2 N to FA, membrane transport and signal transduction, carbohydrate metabolism, and amino acid metabolism play important roles in the gut microbiota (Figure 5C). A comparison of the two groups revealed that, in the FA group, most of the microbiota functions were related to carbohydrate metabolism, membrane transport, nucleotide metabolism, signal transduction, replication and repair, and translation, which suggested that FA harbored the most abundant microbial functions (Figure 5D).

**Figure 6:**
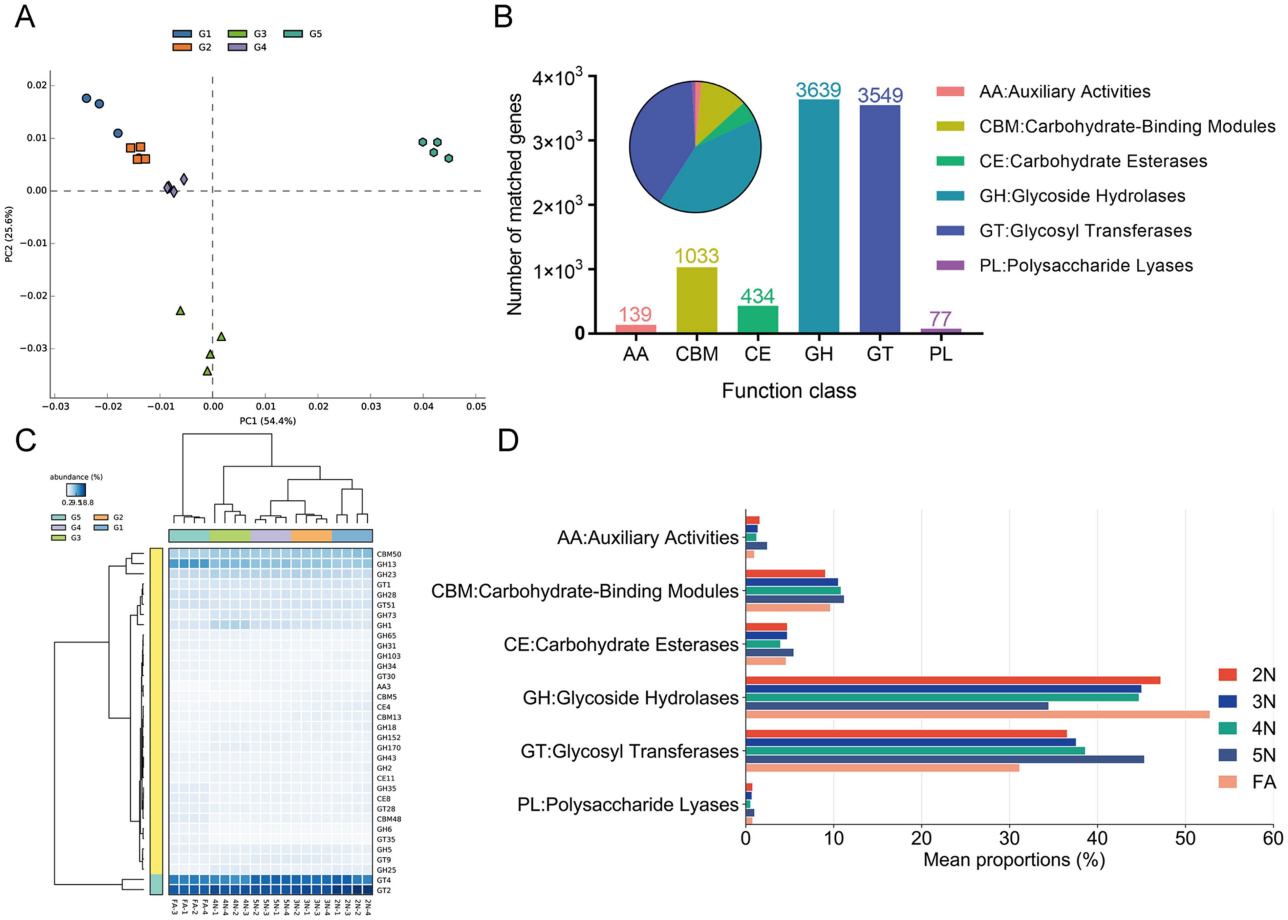
Functional enrichment and comparison of CAZy pathway levels 1 and 2 of the gut microbiome associated with different developmental stages in *R. pedestris*. **(A)** PCA plot based on CAZy pathway level 2 of gut microbiome gene-family abundance. **(B)** Matched gene numbers of CAZy pathway annotations at level 1. **(C)** Relative abundances of CAZy level 2 functions of the gut microbiome during the different developmental stages of *R. pedestris*. **(D)** Relative abundances of CAZy level 1 significantly differed across developmental stages. Different colors represent different groups, and the color scale represents the percentage abundance of a certain CAZy at level 1. 2 N, 2nd instar nymph; 3 N, 3rd instar nymph; 4 N, 4th instar nymph; 5 N, 5th instar nymph; FA, female adult.

Owing to the highest abundance of genes associated with carbohydrate metabolism in the guts of the five groups (Figure 5B), we predicted carbohydrate-active enzymes (CAZy) of the gut microbiota on the basis of the CAZy database to further understand their activity in the gut microbiota. The CAZy (version 2023.03) database is a professional-level database for studying carbohydrate enzymes, covering six main functional categories: glycoside hydrolases (GHs), glycosyl transferases (GTs), polysaccharide lyases (PLs), carbohydrate esterases (CEs), auxiliary activities (AAs), and carbohydrate-binding modules (CBMs). A PCA plot based on gene enzyme function revealed that the samples enriched from 2 N to FA were almost completely spatially separated (Figure 6A). At the first CAZy classification levle, ove 66% genes of GHs (69.6%) and GTs (66.7%) were matched, indicating that these genes play a prominent role in the gut of stinkbugs (Figure 6B). Besides, the genes of CBMs (13.2%) are also essential enzyme families in the gut microbiome of stinkbugs (Figure 6B; Supplementary Table S10). At the second CAZy classification levle, GT4 and GT2 were significantly enriched in the GT family from 2 N to FA. GH13 and CBM50 were also relatively abundant in the gut microbiome of stinkbugs (Figure 6C; Supplementary Table S11). At the third CAZy classification levle, lipopolysaccharide N-acetylglucosaminyltransferase (EC 2.4.1.56) was the most abundant enzyme in five groups (Supplementary Table S12). Notably, the enzyme genes encoding the GH and GT families were significantly more abundant in the gut microbiota of different developmental stages of *R. pedestris* than other CAZy functional classes (Figure 6D). Therefore, GHs and GTs play important roles in the continuous development of stinkbugs.

### 3.5 ARGs abundance analysis of the *Riptortus pedestris* gut microbiota

**Figure 7:**
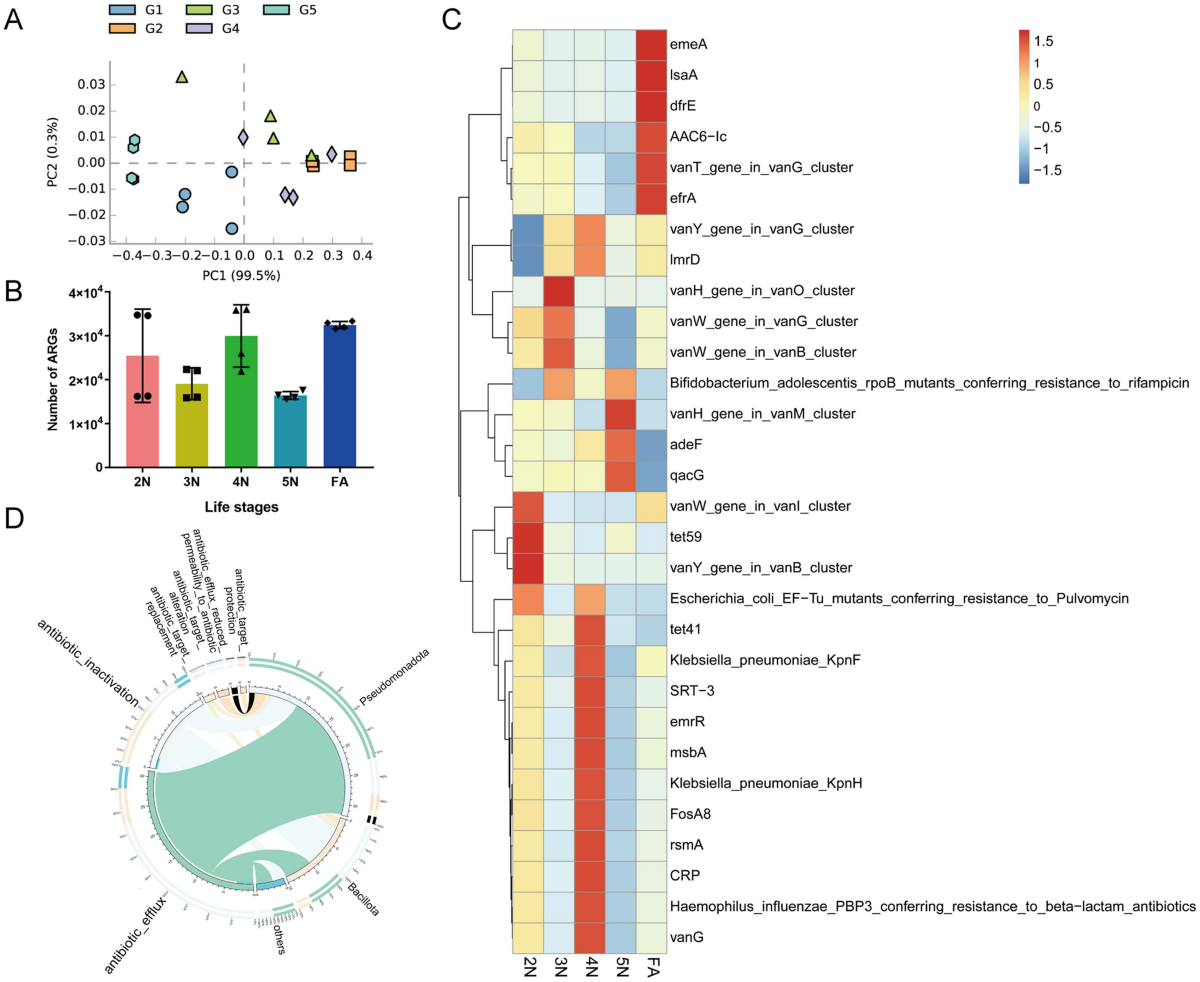
Abundances of antibiotic resistance genes in different developmental stages of *R. pedestris*. **(A)** PCA plot based on the abundance of resistance genes in the gut microbiome. **(B)** Differences in the number of ARGs in different groups. **(C)** Heatmap of the prevalent ARGs in the gut at different developmental stages. **(D)** The mechanism of action of resistance genes and their relationships with species at the phylum level. 2 N, 2nd instar nymph; 3 N, 3rd instar nymph; 4 N, 4th instar nymph; 5 N, 5th instar nymph; FA, female adult.

Annotate the gut microbiome based on the CARD database, we further explored the relationship between antibiotic genotypes and phenotypes in different age groups of the stinkbug. The PCA plot based on ARG abundance revealed that almost all of the samples from G1 to G5 were spatially separated (Figure 7A). In different developmental stages of *R. pedestris*, ARGs were extremely prevalent and exceeded 16,000 (Figure 7B). A total of 47 resistance genes were predicted from 2 N to 5 N (Supplementary Table S13). The *tet59* gene encoding tetracycline and the *vanY* gene encoding D, D-carboxypeptidase antibiotic resistance were most prevalent in the gut microbiota of 2 N (Figure 7C). The *vanH* gene, encoding the lactate dehydrogenase antibiotic resistance gene, was most abundant in the gut microbiome of 3 N. The types of ARGs including *vanG, CRP, rsmA, FosA8, KpnH, msbA, emrR, SRT-3, KpnF, tet41, ImrD,* and *vanY* were the most diverse in the gut microbiota of 4 N. The ARGs including *vanH*, *adeF*, and *qacG* were more abundant in the gut microbiota of 5 N. The ARGs including *emeA*, *IsaA*, *dfrE, AAC6-I*c, *vanT*, and *efrA* were more abundant in the gut microbiota of FA (Figure 7C). Our results revealed a close relationship between the gut microbiota and ARGs.

The mechanisms of antibiotic resistance in bacteria include antibiotic efflux, antibiotic inactivation, antibiotic target alteration, antibiotic target protection, antibiotic target replacement and reduced permeability to antibiotics. We conducted a correlation analysis between the richness of bacteria and ARGs. Among the *R. pedestris* gut microbiota, Pseudomonadota and Bacillota were the principal phyla related to ARGs (Figure 7D). The bacteria from Pseudomonadota were associated with approximately 32 ARGs, particularly antibiotic efflux genes. Bacteria from Bacillota are associated with approximately 15 ARGs, especially antibiotic efflux genes and antibiotic inactivation genes (Supplementary Table S14).

### 3.6 Gut bacteria play important roles in the development of *Riptortus pedestris*

To identify the effects of gut bacteria on the development of *R. pedestris*, the relative abundance of *Burkholderia*, *Caballeronia*, *Enterococcus* and *Serratia*, the number of molted insects and the life span of the insects were detected and analysed. The low *Burkholderia* titre line (–BRp) was generated by treatment with tetracycline hydrochloride at 1 mg/mL (Figure 2C, *p* < 0.01), whereas the other two treatments did not significantly affect the *Burkholderia* titre. The *Burkholderia* titre was reduced by 75% in the –BRp stinkbug group compared with the +BRp stinkbug group (Figure 2C). Likewise, Compared with the treatment without tetracycline hydrochloride (CK), titre of *Caballeronia* was significantly diminished by 74% by treatment with tetracycline hydrochloride at 1 mg/mL (Treatment) (Figure 2D, *p* < 0.05), however the titre of *Enterococcus* and *Serratia* were not significant changes between CK and treatment. We found that the molt number of the treated nymphs was significantly lower in the –BRp group than in the +BRp stinkbug group (Figure 2E, *p* < 0.001). Moreover, under the –BRp, 74EF, E75, and Er treatments, three ecdysteroid-related genes were downregulated. Among them, the expression of 74EF and E75 was significantly downregulated (Figure 2F). The comparison of the developmental periods of the –BRp and + BRp stinkbugs revealed that the –BRp stinkbug remained the 2nd nymph until it died on the 12th day, whereas the +BRp stinkbug successfully developed into an adult (Figure 2G). Taken together, our data demonstrated that low titres of *Burkholderia* strongly affected the development of *R. pedestris*, indicating their importance in the host gut.

## 4 Discussion

Soybeans are important grain crops in China, and persistent symptoms caused by stinkbugs strongly affect the yield and quality of soybeans. Therefore, the prevention and control of stinkbugs has become a research hotspot in soybean production. The gut microbiota affects hosts in multiple ways, including immunity, development, and drug resistance. The developmental cycle of the stinkbug includes eggs, 1st–5th instar nymphs, and adults. In previous studies, three gut bacteria, *Burkholderia*, *Caballeronia insecticola*, and *Serratia marcescens*, have been researched deeply from multiple perspectives (Takeshita et al., 2014; Lee and Lee, 2022; Jouan et al., 2024), but further research is needed on other bacteria. Our study revealed the gut microbial abundance and diversity across 2nd–5th instar nymphs of *R. pedestris*. In this study, we found that *Enterococcus* and *Serratia* were the dominant genera in the five groups. Moreover, *Caballeronia* had the highest proportion in the 5 N groups (Figure 4). Intriguingly, at the species level, *Caballeronia* sp. presented the greatest number of species in the 5 N.

In contrast, *Enterococcus faecalis* and *Serratia marcescens* presented the greatest distributions in the other four groups, especially in female adults (Figures 4C,D). *Caballeronia* sp., *Enterococcus faecalis* and *Serratia marcescens* have been reported to have close relationships with their hosts and play important roles in host immunity, diet, and physiology (Ohbayashi et al., 2022; Zhang X. et al., 2022; Hu et al., 2024). For example, *Enterococcus faecalis* can produce various antibacterial substances that have good inhibitory effects on common pathogenic bacteria in animals (Zhang X. et al., 2022). The genus *Burkholderia* has been proven to be a dominant symbiotic bacterium in both plant and insect intestines (Bernabeu et al., 2015; Kikuchi et al., 2020), and *Caballeronia* sp. is one of the members of this genus (Takeshita and Kikuchi, 2020).

Furthermore, previous studies have shown that *Burkholderia* accounts for 90% of the microbial composition in the fourth midgut section of *R. pedestris* from distinct geographical fields in China (Shan et al., 2024). What’s more, *Caballeronia* sp. also resides in the fourth midgut section of *R. pedestris* (Jouan et al., 2024). Therefore, *Caballeronia* sp. and *Burkholderia* sp. may play important roles in the growth and development of *R. pedestris* nymphs and adults. After *Burkholderia* titres were significantly reduced in *R. pedestris*-fed antibiotics, molting decreased, the developmental duration was delayed, and the lifespan was shortened (Figures 2C,E–G). Hence, the gut symbiont *Burkholderia i*s very important in the different developmental stages of *R. pedestris*, but the richness of *Burkholderia* fluctuates at different stages (Supplementary Figure S4). During the development of stinkbugs, they undergo multiple transformations, and most of the microbial community on the feeding membrane is lost during each molt. However, the presence of special crypt structures provides a relatively stable environment for intestinal microbiota colonization, thereby promoting microbial retention.

The alpha diversity results revealed that there are many unknown intestinal bacteria in female insects, and their functions need to be further explored and studied. More gut microbes were identified in 3rd- and 4th-instar nymphs than in the other stages (Figures 3E,F). Krona visualization analyses (Ondov et al., 2011) revealed that 36% of the female adults were unassigned bacteria; however, in the other stages, 9, 8, 11 and 2% were unassigned bacteria, respectively (Supplementary Figures S2A–E). Therefore, unknown bacteria need further in-depth study.

The diversity of the gut microbiota has different effects on the host. On the one hand, the gut microbiota plays an important role in improving host food utilization and synthesizing necessary nutrients. On the other hand, the interactions between the gut microbiota and the external environment, as well as between host insects, also demonstrate the impact of the gut microbiota on host behavior in multiple ways. *Bifidobacterium* and *Gilliamella* in the bee digestive system can assist the host in metabolizing hemicellulose and pectin in pollen (Zheng et al., 2019). *Enterococcus* and *Pseudomonas* play key roles in the degradation of polymers in the gut of *Spodoptera litura* (Xia et al., 2020). Our study also demonstrated that most gut bacteria participate in nutrition and energy metabolism, as well as in environmental information processing (Figure 5). Carbohydrate enzymes not only directly participate in the digestion and absorption of food in insects, but also promote the growth, development, and survival of insects through interactions with the gut microbiota. For *R. pedestris*, 5th-instar nymphs and female adults need more carbohydrate-related enzymes (Figure 6) to digest food so that they can provide more nutrients and energy.

ARGs play an important role in tolerance to antibiotics. These genes encode antibiotic resistance genes, which enable insects to resist the toxicity of antibiotics and protect them from the negative effects of antibiotics (Fitzpatrick and Walsh, 2016). In addition, as an important component of the ecosystem, insects may transmit antibiotic resistance genes in their intestines through the food chain, which can have an impact on human health (Rawat et al., 2023). In this study, the genus *Enterococcus* accounted for a large proportion of the *R. pedestris* in the gut. A previous study revealed that the abundance of *Enterococcus* was closely related to glycopeptide resistance from *vanY* and *vanX* (Arthur et al., 1998). The use of vancomycin antibiotics from actinomycetes may have caused a high abundance of *vanR* in the guts of different developmental stages of *R. pedestris.* In this study, many glycopeptide ARGs, such as vanG, vanH, vanW, and vanY, in the gut of insect antibiotic resistance genes from actinomycetes were detected in the gut of soybean *stinkbugs* (Figure 7C). Therefore, many ARGs in the gut of *R. pedestris* may be strongly related to the frequent use of antibiotics. In addition, the resistance mechanisms of these resistance genes deserve further investigation to clarify the drug resistance mechanism of *R. pedestris*.

## 5 Conclusion

In conclusion, we analysed the composition and diversity of the gut microbiota between 2nd–5th-year nymphs and female adults of *R. pedestris* and reported that *Enterococcus* and *Caballeronia* were the most dominant genera in the 2 N–5 N stages, whereas *Enterococcus* and *Serrtia* were dominant in the 4 N and FA stages. Moreover*, Burkholderia–Paraburkholderia–Caballeronia* was the most abundant genus in the 5 N stage. Furthermore, antibiotic treatment reduced molting, delayed development, and shortened the lifespan of *R. pedestris*. We also determined that the frequent use of antibiotics caused severe antibiotic resistance in the gut of *R. pedestris*. According to our results, monitoring the antibiotic resistance of bacteria from Bacillota and Pseudomonadota in the *R. pedestris* gut environment is essential to reduce the transmission risk from ARGs and drug-resistant bacteria. Overall, these research results provide an important basis for further exploration of the role of symbiotic bacteria in the gut of stink bugs and the application of important microorganisms and ARGs in the prevention and control of stink bugs.

## Data Availability

The raw sequence data reported in this paper have been deposited in the Genome Sequence Archive (Chen et al., 2021), in National Genomics Data Center (Xue et al., 2022), China National Center for Bioinformation/Beijing Institute of Genomics, Chinese Academy of Sciences (https://ngdc.cncb.ac.cn/gsa), GSA accession number CRA019496.

## References

[ref1] Arthur M. Depardieu F. Cabanié L. Reynolds P. Courvalin P. (1998). Requirement of the VanY and VanX D,D-peptidases for glycopeptide resistance in enterococci. Mol. Microbiol. 30, 819–830. doi: 10.1046/j.1365-2958.1998.01114.x, PMID: 10094630

[ref2] Bernabeu P. R. Pistorio M. Torres-Tejerizo G. Estrada-de P. Galar M. Boiardi J.. (2015). Colonization and plant growth-promotion of tomato by *Burkholderia tropica*. Sci. Hortic. 191, 113–120. doi: 10.1016/j.scienta.2015.05.014

[ref3] Chen T. Chen X. Zhang S. Zhu J. Tang B. Wang A.. (2021). The genome sequence archive family: toward explosive data growth and diverse data types. Genomics Proteomics Bioinformatics 19, 578–583. doi: 10.1016/j.gpb.2021.08.001, PMID: 34400360 PMC9039563

[ref4] Courvalin P. (2006). Vancomycin resistance in gram-positive cocci downloaded from. Clin. Infect. Dis. 42, S25–S34. doi: 10.1086/491711, PMID: 16323116

[ref5] Elston K. M. Maeda G. P. Perreau J. Barrick J. E. (2023). Addressing the challenges of symbiont-mediated RNAi in aphids. PeerJ 11, e14961–e14926. doi: 10.7717/peerj.14961, PMID: 36874963 PMC9983426

[ref6] Engel P. Moran N. A. (2013). The gut microbiota of insects – diversity in structure and function. FEMS Microbiol. Rev. 37, 699–735. doi: 10.1111/1574-6976.12025, PMID: 23692388

[ref7] Feng Q. Liang S. Jia H. Stadlmayr A. Tang L. Lan Z.. (2015). Gut microbiome development along the colorectal adenoma-carcinoma sequence. Nat. Commun. 6:6528. doi: 10.1038/ncomms7528, PMID: 25758642

[ref8] Fitzpatrick D. Walsh F. (2016). Antibiotic resistance genes across a wide variety of metagenomes. FEMS Microbiol. Ecol. 92, 1–8. doi: 10.1093/femsec/fiv168, PMID: 26738556

[ref9] Fu L. Niu B. Zhu Z. Wu S. Li W. (2012). CD-HIT: accelerated for clustering the next-generation sequencing data. Bioinformatics 28, 3150–3152. doi: 10.1093/bioinformatics/bts565, PMID: 23060610 PMC3516142

[ref10] Fu J. Wang J. Huang X. Guan B. Feng Q. Deng H. (2023). Composition and diversity of gut microbiota across developmental stages of Spodoptera frugiperda and its effect on the reproduction. Front. Microbiol. 14, 1–12. doi: 10.3389/fmicb.2023.1237684, PMID: 37789854 PMC10543693

[ref11] Fung C. Rusling M. Lampeter T. Love C. Karim A. Bongiorno C.. (2021). Automation of QIIME2 metagenomic analysis platform. Curr. Protoc. 1:e254. doi: 10.1002/cpz1.254, PMID: 34554657

[ref12] Gao H. Bai L. Jiang Y. Huang W. Wang L. Li S.. (2021). A natural symbiotic bacterium drives mosquito refractoriness to plasmodium infection via secretion of an antimalarial lipase. Nat. Microbiol. 6, 806–817. doi: 10.1038/s41564-021-00899-8, PMID: 33958765 PMC9793891

[ref13] Hu W. Zhao C. Zheng R. Duan S. Lu Z. Zhang Z.. (2024). *Serratia marcescens* induces apoptosis in *Diaphorina citri* gut cells via reactive oxygen species-mediated oxidative stress. Pest Manag. Sci. 80, 602–612. doi: 10.1002/ps.7787, PMID: 37740936

[ref14] Jang S. Kikuchi Y. (2020). Impact of the insect gut microbiota on ecology, evolution, and industry. Curr. Opin. Insect Sci. 41, 33–39. doi: 10.1016/j.cois.2020.06.004, PMID: 32634703

[ref15] Jia B. Raphenya A. R. Alcock B. Waglechner N. Guo P. Tsang K. K.. (2017). CARD 2017: expansion and model-centric curation of the comprehensive antibiotic resistance database. Nucleic Acids Res. 45, D566–D573. doi: 10.1093/nar/gkw1004, PMID: 27789705 PMC5210516

[ref16] Jouan R. Lextrait G. Lachat J. Yokota A. Cossard R. Naquin D.. (2024). Transposon sequencing reveals the essential gene set and genes enabling gut symbiosis in the insect symbiont Caballeronia insecticola. ISME Commun. 4, 1–13. doi: 10.1093/ismeco/ycad001, PMID: 38282642 PMC10809759

[ref17] Karlsson F. H. Fåk F. Nookaew I. Tremaroli V. Fagerberg B. Petranovic D.. (2012). Symptomatic atherosclerosis is associated with an altered gut metagenome. Nat. Commun. 3:1245. doi: 10.1038/ncomms2266, PMID: 23212374 PMC3538954

[ref18] Karlsson F. H. Tremaroli V. Nookaew I. Bergström G. Behre C. J. Fagerberg B.. (2013). Gut metagenome in European women with normal, impaired and diabetic glucose control. Nature 498, 99–103. doi: 10.1038/nature12198, PMID: 23719380

[ref19] Kikuchi Y. Hayatsu M. Hosokawa T. Nagayama A. Tago K. Fukatsu T. (2012). Symbiont-mediated insecticide resistance. Proc. Natl. Acad. Sci. USA 109, 8618–8622. doi: 10.1073/pnas.1200231109, PMID: 22529384 PMC3365206

[ref20] Kikuchi Y. Hosokawa T. Fukatsu T. (2011). Specific developmental window for establishment of an insect-microbe gut symbiosis. Appl. Environ. Microbiol. 77, 4075–4081. doi: 10.1128/AEM.00358-11, PMID: 21531836 PMC3131632

[ref21] Kikuchi Y. Meng X. Y. Fukatsu T. (2005). Gut symbiotic bacteria of the genus Burkholderia in the broad-headed bugs Riptortus clavatus and *Leptocorisa chinensis* (Heteroptera: Alydidae). Appl. Environ. Microbiol. 71, 4035–4043. doi: 10.1128/AEM.71.7.4035-4043.2005, PMID: 16000818 PMC1169019

[ref22] Kikuchi Y. Ohbayashi T. Jang S. Mergaert P. (2020). Burkholderia insecticola triggers midgut closure in the bean bug *Riptortus pedestris* to prevent secondary bacterial infections of midgut crypts. ISME J. 14, 1627–1638. doi: 10.1038/s41396-020-0633-3, PMID: 32203122 PMC7305115

[ref23] Kim J. K. Lee J. B. Huh Y. R. Jang H. A. Kim C. H. Yoo J. W.. (2015a). Burkholderia gut symbionts enhance the innate immunity of host *Riptortus pedestris*. Dev. Comp. Immunol. 53, 265–269. doi: 10.1016/j.dci.2015.07.006, PMID: 26164198

[ref24] Kim J. K. Son D. W. Kim C. H. Cho J. H. Marchetti R. Silipo A.. (2015b). Insect gut symbiont susceptibility to host antimicrobial peptides caused by alteration of the bacterial cell envelope. J. Biol. Chem. 290, 21042–21053. doi: 10.1074/jbc.M115.651158, PMID: 26116716 PMC4543662

[ref25] Lee J. Lee D. W. (2022). Insecticidal Serralysin of *Serratia marcescens* is detoxified in M3 midgut region of *Riptortus pedestris*. Front. Microbiol. 13:913113. doi: 10.3389/fmicb.2022.913113, PMID: 35711769 PMC9197470

[ref26] Li D. Liu C. M. Luo R. Sadakane K. Lam T. W. (2015). MEGAHIT: an ultra-fast single-node solution for large and complex metagenomics assembly via succinct de Bruijn graph. Bioinformatics 31, 1674–1676. doi: 10.1093/bioinformatics/btv033 25609793

[ref27] Li K. Zhang X. Guo J. Penn H. Wu T. Li L.. (2019). Feeding of *Riptortus pedestris* on soybean plants, the primary cause of soybean staygreen syndrome in the Huang-Huai-Hai river basin. Crop J. 7, 360–367. doi: 10.1016/j.cj.2018.07.008

[ref28] Luan J. B. Chen W. Hasegawa D. K. Simmons A. Wintermantel W. M. Ling K. S.. (2015). Metabolic coevolution in the bacterial symbiosis of whiteflies and related plant sap-feeding insects. Genome Biol. Evol. 7, 2635–2647. doi: 10.1093/gbe/evv170, PMID: 26377567 PMC4607527

[ref29] Luo J. Cheng Y. Guo L. Wang A. Lu M. Xu L. (2021). Variation of gut microbiota caused by an imbalance diet is detrimental to bugs’ survival. Sci. Total Environ. 771:144880. doi: 10.1016/j.scitotenv.2020.144880 33736123

[ref30] Martínez J. L. Coque T. M. Baquero F. (2015). What is a resistance gene? Ranking risk in resistomes. Nat. Rev. Microbiol. 13, 116–123. doi: 10.1038/nrmicro3399, PMID: 25534811

[ref31] Nakajima Y. Sakuma M. Sasaki R. Fujisaki K. (2010). Adaptive traits of *riptortus pedestris* nymphs (Heteroptera: Alydidae) for locating host plants. Ann. Entomol. Soc. Am. 103, 439–448. doi: 10.1603/AN09144

[ref32] Ng S. H. Stat M. Bunce M. Simmons L. W. (2018). The influence of diet and environment on the gut microbial community of field crickets. Ecol. Evol. 8, 4704–4720. doi: 10.1002/ece3.3977, PMID: 29760910 PMC5938447

[ref33] Ohbayashi T. Cossard R. Lextrait G. Hosokawa T. Lesieur V. Takeshita K.. (2022). Intercontinental diversity of Caballeronia gut symbionts in the conifer Pest bug *Leptoglossus occidentalis*. Microbes Environ. 37, 1–9. doi: 10.1264/jsme2.ME22042, PMID: 35965097 PMC9530724

[ref34] Ondov B. D. Bergman N. H. Phillippy A. M. (2011). Interactive metagenomic visualization in a web browser. BMC Bioinformatics 12:385. doi: 10.1186/1471-2105-12-385, PMID: 21961884 PMC3190407

[ref35] Preuß A. Röder M. Röder B. (2019). Mosquito larvae control by photodynamic inactivation of their intestinal flora-a proof of principal study on: Chaoborus sp. Photochem. Photobiol. Sci. 18, 2374–2380. doi: 10.1039/c9pp00156e, PMID: 31380867

[ref36] Rawat N. Anjali Shreyata Sabu B. Jamwal R. Devi P.. (2023). Understanding the role of insects in the acquisition and transmission of antibiotic resistance. Sci. Total Environ. 858:159805. doi: 10.1016/j.scitotenv.2022.159805, PMID: 36461578

[ref37] Scher J. U. Sczesnak A. Longman R. S. Segata N. Ubeda C. Bielski C.. (2013). Expansion of intestinal *Prevotella copri* correlates with enhanced susceptibility to arthritis. eLife 2, 1–20. doi: 10.7554/elife.01202, PMID: 24192039 PMC3816614

[ref38] Schmittgen T. D. Livak K. J. (2008). Analyzing real-time PCR data by the comparative CT method. Nat. Protoc. 3, 1101–1108. doi: 10.1038/nprot.2008.73, PMID: 18546601

[ref39] Schoeler M. Caesar R. (2019). Dietary lipids, gut microbiota and lipid metabolism. Rev. Endocr. Metab. Disord. 20, 461–472. doi: 10.1007/s11154-019-09512-0, PMID: 31707624 PMC6938793

[ref40] Segata N. Izard J. Waldron L. Gevers D. Miropolsky L. Garrett W. S.. (2011). Metagenomic biomarker discovery and explanation. Genome Biol. 12:R60. doi: 10.1186/gb-2011-12-6-r60, PMID: 21702898 PMC3218848

[ref41] Sekirov I. Russell S. L. Antunes C. M. Finlay B. (2010). Gut microbiota in health and disease. Physiol. Rev. 90, 859–904. doi: 10.1152/physrev.00045.2009, PMID: 20664075

[ref42] Shamjana U. Vasu D. A. Hembrom P. S. Nayak K. Grace T. (2024). The role of insect gut microbiota in host fitness, detoxification and nutrient supplementation. Antonie van Leeuwenhoek 117, 71–29. doi: 10.1007/s10482-024-01970-0, PMID: 38668783

[ref43] Shan H. W. Xia X. J. Feng Y. L. Wu W. Li H. J. Sun Z. T.. (2024). The plant-sucking insect selects assembly of the gut microbiota from environment to enhance host reproduction. NPJ Biofilms Microbiomes 10:64. doi: 10.1038/s41522-024-00539-z, PMID: 39080326 PMC11289440

[ref44] Shao Y. Chen B. Sun C. Ishida K. Hertweck C. Boland W. (2017). Symbiont-derived antimicrobials contribute to the control of the lepidopteran gut microbiota. Cell Chem. Biol. 24, 66–75. doi: 10.1016/j.chembiol.2016.11.015, PMID: 28107652

[ref45] Sunagawa S. Coelho L. Chaffron S. Kultima J. Labadie K. Salazar G.. (2015). Structure and function of the global ocean microbiome. Science 348:1261359. doi: 10.1126/science.1261359, PMID: 25999513

[ref46] Takeshita K. Kikuchi Y. (2020). Genomic comparison of insect gut symbionts from divergent burkholderia subclades. Genes (Basel) 11, 1–21. doi: 10.3390/genes11070744, PMID: 32635398 PMC7397029

[ref47] Takeshita K. Shibata T. F. Nikoh N. Nishiyama T. Hasebe M. Fukatsu T. (2014). Whole-genome sequence of Burkholderia sp. strain RPE67, a bacterial gut symbiont of the bean bug Riptortus pedestris. Genome Announc. 2, 2–3. doi: 10.1128/genomeA.00556-14.Copyright PMC4064023 24948758

[ref48] Wei Z. Guo W. Jiang S. Yan D. Shi Y. Wu B.. (2023). Transcriptional profiling reveals a critical role of GmFT2a in soybean staygreen syndrome caused by the pest *Riptortus pedestris*. New Phytol. 237, 1876–1890. doi: 10.1111/nph.18628, PMID: 36404128

[ref49] Xia X. Lan B. Tao X. Lin J. You M. (2020). Characterization of *Spodoptera litura* gut Bacteria and their role in feeding and growth of the host. Front. Microbiol. 11, 1–14. doi: 10.3389/fmicb.2020.01492, PMID: 32714311 PMC7344319

[ref50] Xue Y. Bao Y. Zhang Z. Zhao W. Xiao J. He S.. (2022). Database resources of the National Genomics Data Center, China National Center for bioinformation in 2022. Nucleic Acids Res. 50, D27–D38. doi: 10.1093/nar/gkab951, PMID: 34718731 PMC8728233

[ref51] Yan Y. Xu J. Huang W. Fan Y. Li Z. Tian M.. (2024). Metagenomic and Culturomics analysis of microbial communities within surface sediments and the prevalence of antibiotic resistance genes in a Pristine River: the Zaqu River in the Lancang River source region, China. Microorganisms 12, 1–23. doi: 10.3390/microorganisms12050911 PMC11124135 38792738

[ref52] Yang S. Liu Y. Yang N. Lan Y. Lan W. Feng J.. (2022). The gut microbiome and antibiotic resistome of chronic diarrhea rhesus macaques (*Macaca mulatta*) and its similarity to the human gut microbiome. Microbiome 10, 29–13. doi: 10.1186/s40168-021-01218-3, PMID: 35139923 PMC8827259

[ref53] Yun J. H. Roh S. W. Whon T. W. Jung M. J. Kim M. S. Park D. S.. (2014). Insect gut bacterial diversity determined by environmental habitat, diet, developmental stage, and phylogeny of host. Appl. Environ. Microbiol. 80, 5254–5264. doi: 10.1128/AEM.01226-14, PMID: 24928884 PMC4136111

[ref54] Zeller G. Tap J. Voigt A. Y. Sunagawa S. Kultima J. R. Costea P. I.. (2014). Potential of fecal microbiota for early-stage detection of colorectal cancer. Mol. Syst. Biol. 10, 766–718. doi: 10.15252/msb.20145645, PMID: 25432777 PMC4299606

[ref55] Zhang X. Feng H. He J. Liang X. Zhang N. Shao Y.. (2022). The gut commensal bacterium *Enterococcus faecalis* LX10 contributes to defending against Nosema bombycis infection in *Bombyx mori*. Pest Manag. Sci. 78, 2215–2227. doi: 10.1002/ps.6846, PMID: 35192238 PMC9314687

[ref56] Zhang Z. Mu X. Cao Q. Shi Y. Hu X. Zheng H. (2022). Honeybee gut Lactobacillus modulates host learning and memory behaviors via regulating tryptophan metabolism. Nat. Commun. 13, 2037–2013. doi: 10.1038/s41467-022-29760-0, PMID: 35440638 PMC9018956

[ref57] Zhang H. Wang Y. Wang Z. Ding W. Xu K. Li L.. (2022). Modelling the current and future potential distribution of the bean bug *Riptortus pedestris* with increasingly serious damage to soybean. Pest Manag. Sci. 78, 4340–4352. doi: 10.1002/ps.7053, PMID: 35754391

[ref58] Zhang Y. Zhang S. Xu L. (2023). The pivotal roles of gut microbiota in insect plant interactions for sustainable pest management. NPJ Biofilms Microbiomes 9:66. doi: 10.1038/s41522-023-00435-y, PMID: 37735530 PMC10514296

[ref59] Zheng H. Perreau J. Powell J. Han B. Zhang Z. Kwong W. K.. (2019). Division of labor in honey bee gut microbiota for plant polysaccharide digestion. Proc. Natl. Acad. Sci. USA 116, 25909–25916. doi: 10.1073/pnas.1916224116, PMID: 31776248 PMC6926048

