# Supplementary material for: The intestinal microbial community and function of Riptortus pedestris at different developmental stages and its effects on development
Source: Front Microbiol. 2025 Jan 22;16:1517280. doi: 10.3389/fmicb.2025.1517280 (PMC11813222; doi:10.3389/fmicb.2025.1517280)
Supplement: Supplementary file 1 [file Supplementary_file_1.docx]

**SU****PPLEMENTARY MATERIAL**

**Table S1: Primers used in this study.**

**Table S2: Sequencing data of intestinal microbial metagenomics in different groups of *Riptortus pedestris.***

**Table S3: Alpha diversity analysis (mean ± SEM) of the gut microbiota at different developmental stages in *Riptortus pedestris.***

**Figure S1: Correlation analysis between samples on the basis of gene number.** 2 N, 2nd instar nymph; 3 N, 3rd instar nymph; 4 N, 4th instar nymph; 5 N, 5th instar nymph; FA, female adult.

**Figure S2: Visualization of the annotation results of the gut microbiota at different developmental stages through Krona.** A-E, 2nd-5th instar nymph and female adult.

**Figure S3: Significant difference analysis of the gut bacterial community across the different groups by LEfSe and heatmap analysis with LDA = 4.** 2 N, 2nd instar nymph; 3 N, 3rd instar nymph; 4 N, 4th instar nymph; 5 N, 5th instar nymph; FA, female adult.

**Figure S4: Comparison of the relative abundance of the gut symbiont *Burkholderia* across the different developmental stages.** 2 N, 2nd instar nymph; 3 N, 3rd instar nymph; 4 N, 4th instar nymph; 5 N, 5th instar nymph; FA, female adult. Significant differences between the different developmental stages are indicated by asterisks: ***, *P* < 0.001.

Table S1

| Primer name | Sequence (5'→3') | Efficiency (%) | Reference |
| --- | --- | --- | --- |
| Burk-F | GTAGGGTGCGAGCGTTAATC | 104.15 | This study |
| Burk-R | AAGCTTGCCAGTCACCAATG |  |  |
| Ca-F | AATACGTTCCCGGGTCTTGT | 106.16 | This study |
| Ca-R | GTCCTCCTTGCGGTTAGACT |  |  |
| En-F | TTCGGCAATGGACGAAAGTC | 90.12 | This study |
| En-R | TAGCCGTGGCTTTCTGGTTA |  |  |
| Se-F | ATTCGATGCAACGCGAAGAA | 89.85 | This study |
| Se-R | AGCACCTGTCTCAGAGTTCC |  |  |
| 74EF-F | TACGTCGCAAGTCTACGTCA | 109.16 | This study |
| 74EF-R | GCATGGTGTTGGCTCAAGAT |  |  |
| E75-E | CGCCATCAGAGGAGTTGTTG | 100.29 | This study |
| E75-R | CCACTGTGGATTGAGTCCCT |  |  |
| Er-F | ACCATTACAATGCGCTCACC | 101.38 | This study |
| Er-R | TTCAGCCTACACTCTTGGCA |  |  |
| EF1α-F | CCTGCATCCGTTGCTTTTGT |  | Kim et al., 2015b |
| EF1α-R | GCATCGAGGGCTTCAATAA |  |  |

**Table S2**

| Group | Sample | RawData (Mb) | CleanData (Mb) | Clean_Q20 (%) | Clean_GC (%) | Effective (%) |
| --- | --- | --- | --- | --- | --- | --- |
| G1 | 2 N-1 | 6321.60 | 6282.04 | 98.50 | 52.22 | 99.37 |
|  | 2 N-2 | 6,127.51 | 6,112.02 | 98.73 | 48.05 | 99.75 |
|  | 2 N-3 | 6,073.59 | 6,054.40 | 98.59 | 52.95 | 99.68 |
|  | 2 N-4 | 6,220.08 | 6,191.34 | 98.65 | 48.49 | 99.54 |
| G2 | 3 N-1 | 6440.17 | 6389.77 | 98.50 | 50.81 | 99.22 |
|  | 3 N-2 | 6,502.28 | 6,475.95 | 98.22 | 52.45 | 99.60 |
|  | 3 N-3 | 6,049.31 | 6,030.28 | 98.59 | 45.69 | 99.69 |
|  | 3 N-4 | 6,509.09 | 6,477.32 | 98.65 | 45.85 | 99.51 |
| G3 | 4 N-1 | 6,632.77 | 6,601.50 | 98.51 | 42.99 | 99.53 |
|  | 4 N-2 | 6606.22 | 6548.58 | 98.41 | 51.91 | 99.13 |
|  | 4 N-3 | 6,333.59 | 6,292.78 | 98.47 | 52.20 | 99.37 |
|  | 4 N-4 | 6,509.33 | 6,478.62 | 98.63 | 43.22 | 99.53 |
| G4 | 5 N-1 | 6,451.49 | 6,419.16 | 98.17 | 57.75 | 99.50 |
|  | 5 N-2 | 6,109.06 | 6,081.24 | 98.28 | 55.21 | 99.55 |
|  | 5 N-3 | 6195.00 | 6140.12 | 98.12 | 56.14 | 99.11 |
|  | 5 N-4 | 6,592.09 | 6,563.56 | 98.10 | 58.82 | 99.57 |
| G5 | FA-1 | 6,350.94 | 6,306.36 | 99.03 | 41.55 | 99.30 |
|  | FA-2 | 6,906.23 | 6,799.76 | 98.78 | 40.89 | 98.46 |
|  | FA-3 | 6,748.17 | 6,630.77 | 98.69 | 41.00 | 98.26 |
|  | FA-4 | 6174.20 | 6114.36 | 98.62 | 41.66 | 99.03 |

**Notes**: Clean_Q20(%): the percentage of bases with a sequencing error rate less than 0.01 (mass value greater than 20) in CleanData

Clean_GC(%): the percentage of G and C bases in CleanData

Effective(%): the percentage of CleanData to RawData

2 N, 2nd instar nymph; 3 N, 3rd instar nymph; 4 N, 4th instar nymph; 5 N, 5th instar nymph; FA, female adult

**Table S3**

| **Sample** | **2 N(n=4)** | **3 N(n=4)** | **4 N(n=4)** | **5 N(n=4)** | **FA(n=4)** |
| --- | --- | --- | --- | --- | --- |
| ACE | 258.01±7.93b | 254.74±7.04bc | 195.44±5.02e | 224.70±1.60d | 905.87±1.96a |
| Chao1 | 260.80±9.62b | 255.88±7.93b | 194.65±4.78b | 224.50±1.66b | 911.45±1.24a |
| Shannon | 2.23±0.13b | 2.62±0.08a | 2.61±0.28ab | 1.67±0.37c | 1.74±0.01c |
| Simpson | 0.63±0.06b | 0.73±0.02a | 0.71±0.07a | 0.41±0.10c | 0.51±0.01c |
| Observes species | 250.50±6.10b | 253.00±7.58b | 194.25±4.55d | 223.75±1.64c | 901.50±3.20a |
| Goods coverage | 1.00±0.00a | 1.00±0.00a | 1.00±0.00a | 1.00±0.00a | 1.00±0.00a |

**Notes**: 2 N, 2nd instar nymph; 3 N, 3rd instar nymph; 4 N, 4th instar nymph; 5 N, 5th instar nymph; FA, female adult. Different letters indicate significant differences (*P* < 0.05).


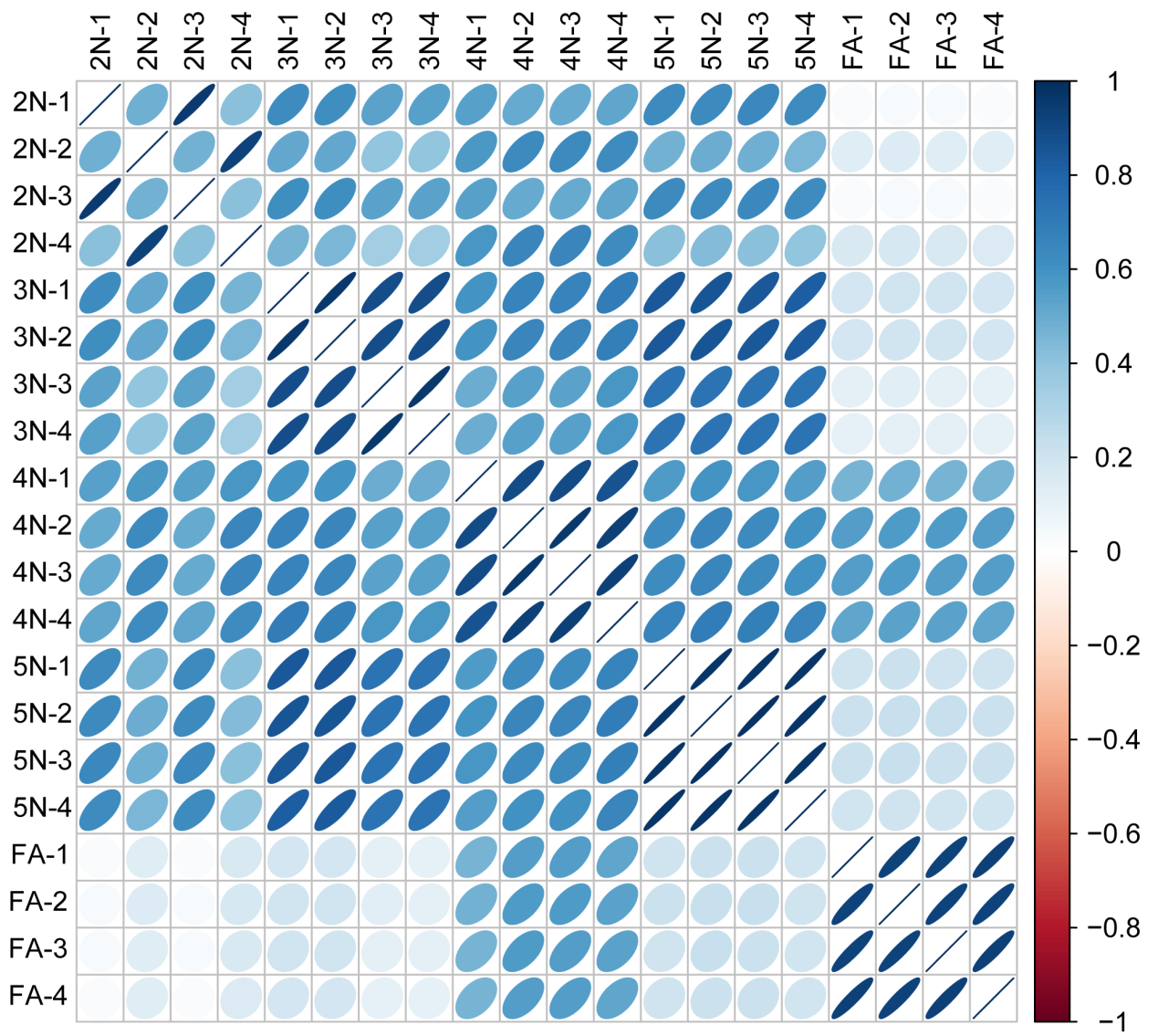


**Figure S1: Correlation analysis between samples on the basis of gene number.** 2 N, 2nd instar nymph; 3 N, 3rd instar nymph; 4 N, 4th instar nymph; 5 N, 5th instar nymph; FA, female adult.

E

D

C

B

A


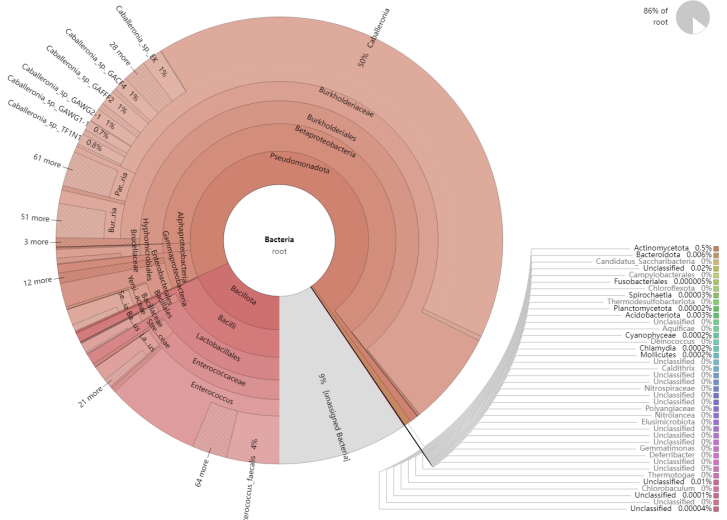

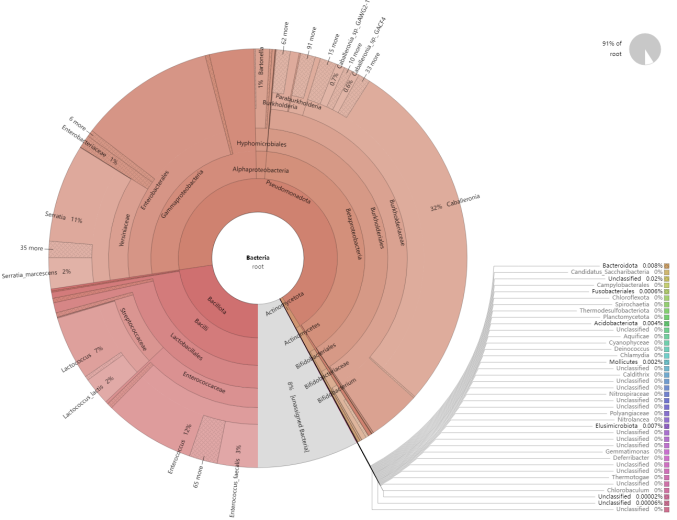

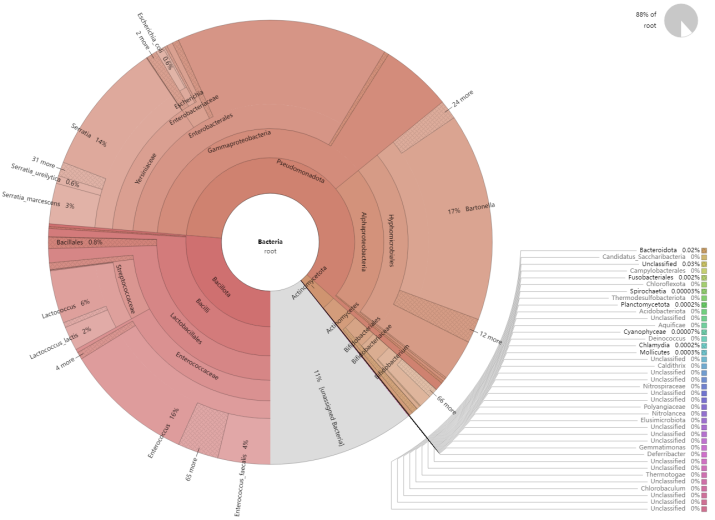

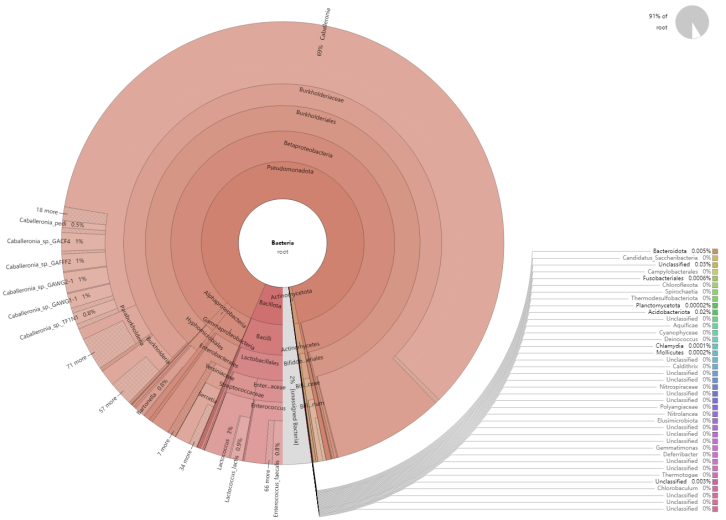

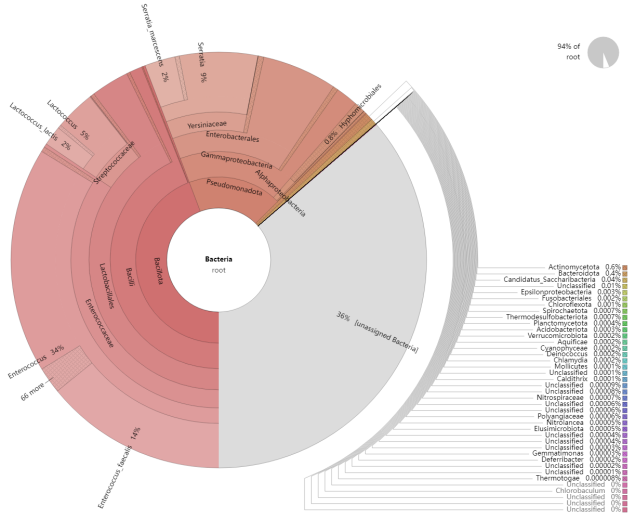


**Figure S2: Visualization of the annotation results of the gut microbiota at different developmental stages through Krona.** A-E, 2nd-5th instar nymph and female adult.


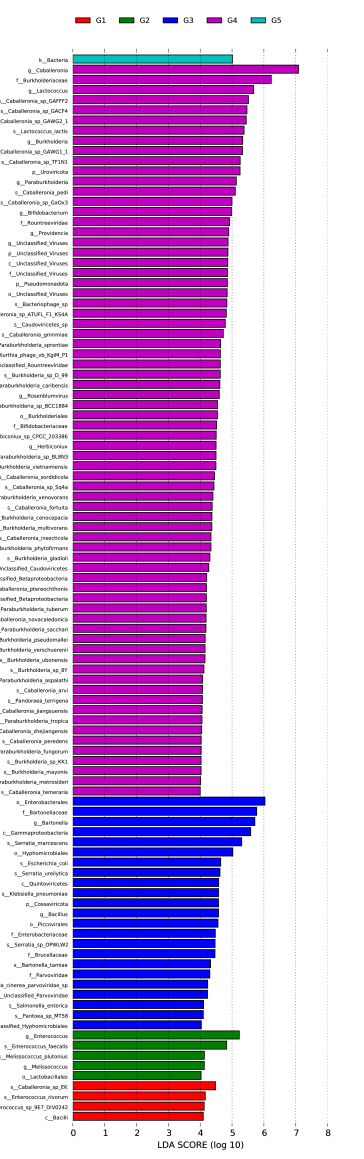

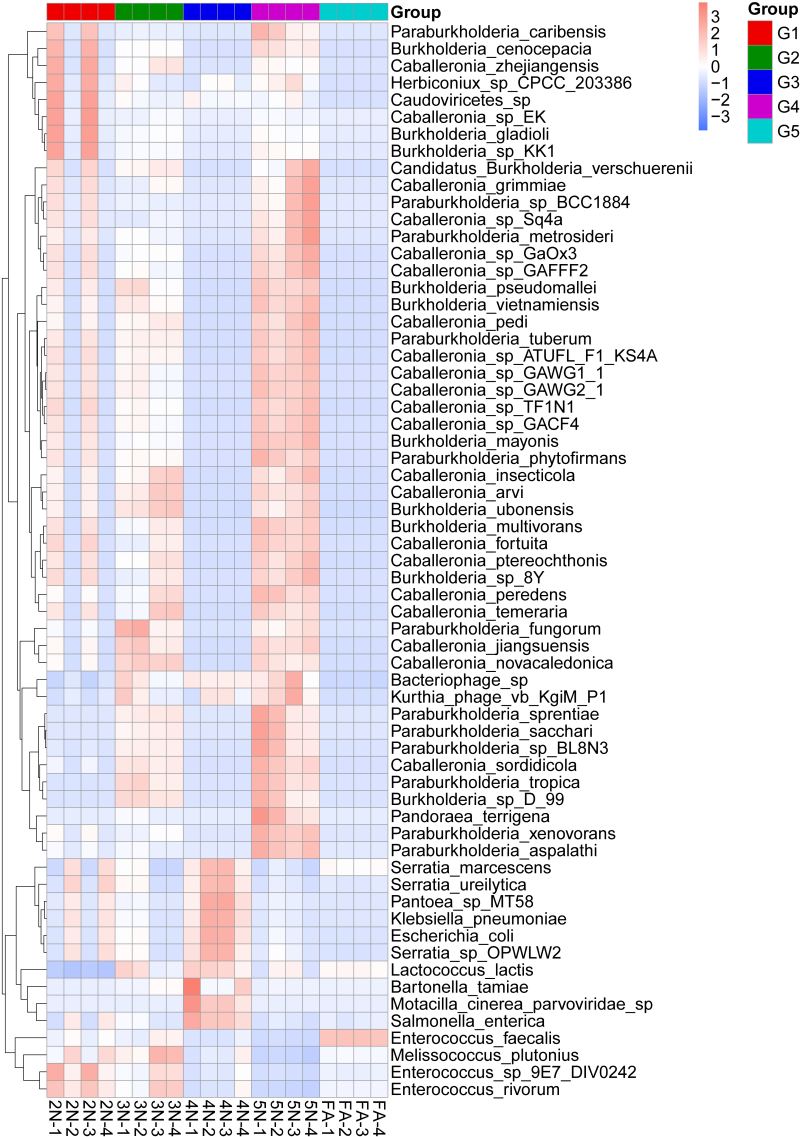


**Figure S3: Significant difference analysis of the gut bacterial community across the different groups by LEfSe and heatmap analysis with LDA** > **4.** 2 N, 2nd instar nymph; 3 N, 3rd instar nymph; 4 N, 4th instar nymph; 5 N, 5th instar nymph; FA, female adult.

**
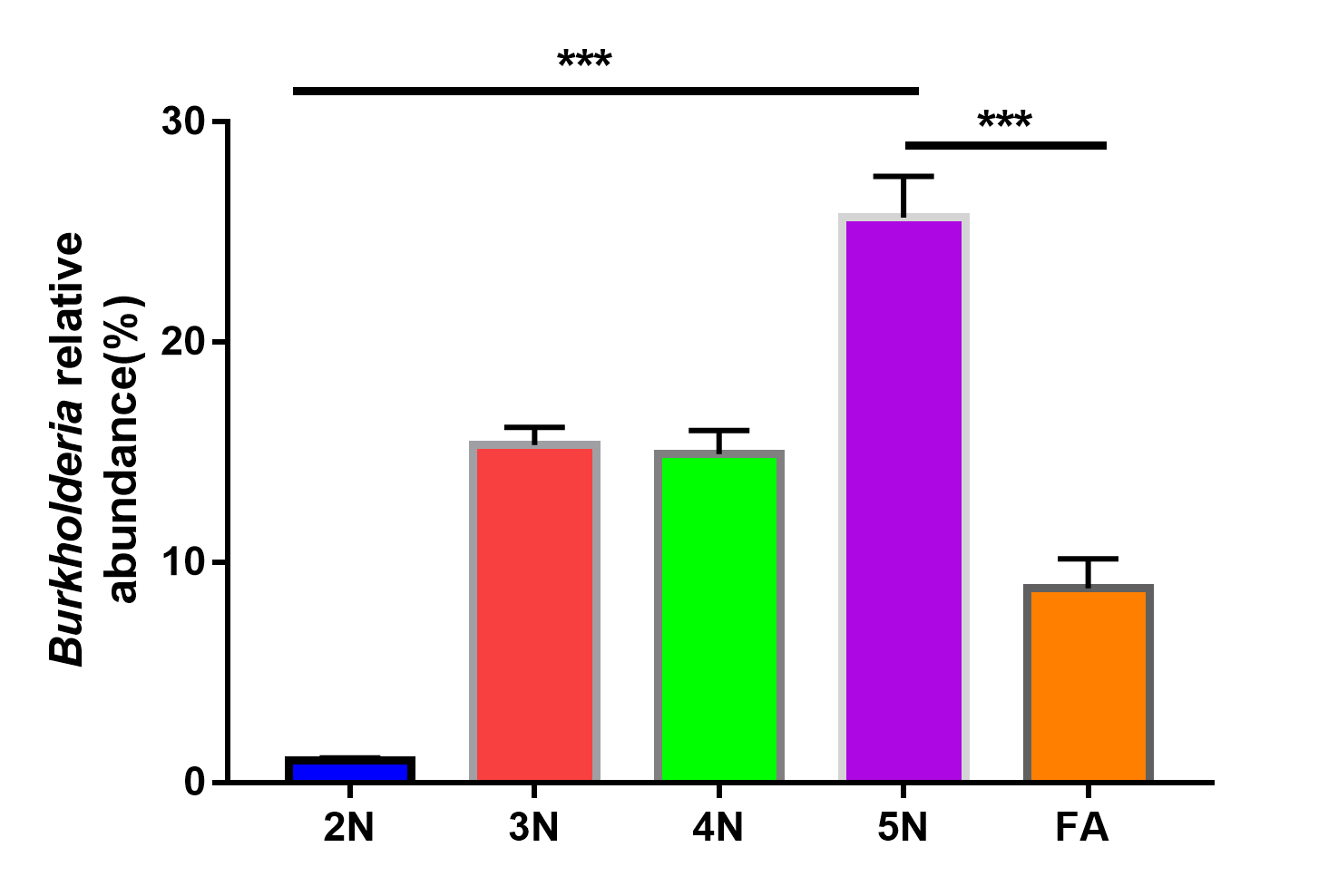
**

**Figure S4: Comparison of the relative abundance of the gut symbiont *Burkholderia* across the different developmental stages.** 2 N, 2nd instar nymph; 3 N, 3rd instar nymph; 4 N, 4th instar nymph; 5 N, 5th instar nymph; FA, female adult. Significant differences between the different developmental stages are indicated by asterisks: ***, *P* < 0.001.
